# Body surface area, height, and body fat percentage as more sensitive risk factors of cancer and cardiovascular disease

**DOI:** 10.1002/cam4.3076

**Published:** 2020-04-27

**Authors:** Shucheng Si, Marlvin A. Tewara, Xiaokang Ji, Yongchao Wang, Yanxun Liu, Xiaoyu Dai, Zhiheng Wang, Fuzhong Xue

**Affiliations:** ^1^ Department of Biostatistics School of Public Health Cheeloo College of Medicine Shandong University Jinan Shandong P.R. China; ^2^ Institute for Medical Dataology Shandong University Jinan P.R. China

**Keywords:** body fat percentage, body surface area, cancer, cardiovascular disease, physical measurements

## Abstract

**Background:**

Limited studies have compared the association between various physical measurements and the risk of cancer or cardiovascular disease (CVD). We aim to explore the best‐individualized indicators of cancer and CVD risk assessment.

**Methods:**

From May 2004 to December 2017, a community‐based cohort in China involving 100 280 participants were enrolled. BMI, height, body surface area (BSA), and body fat percentage (BFP) were compared in parallel about cancer and CVD risk with the multivariable‐adjusted Cox proportional hazard regression model.

**Results:**

Within the follow‐up period, 3107 (3.10%) were diagnosed with cancer and 3721 (3.71%) had CVD. Per‐level increased (in tertile: T1, T2, and T3 level) BSA, height, and BFP was positively associated with the risk of overall cancer [HR (95% CI): 1.10 (1.05‐1.15), 1.12 (1.07‐1.18), and 1.10 (1.03‐1.16), respectively], whereas BMI was insignificant. Compared with the reference group (T2), the highest BSA level (T3) was positively associated with overall cancer incidence for both male [HR (95% CI): 1.28 (1.13‐1.45)] and female [HR (95% CI): 1.13 (1.00‐1.28)]. The BSA, height, and BFP also significantly associated with some site‐specific cancers including thyroid, stomach, breast, urinary system, and skin cancer. Meanwhile, BFP presented a strong positive association with overall CVD [HR (95% CI): 1.22 (1.15‐1.30) in trend] in both gender and associated with nearly all CVD subtypes especially the myocardial infarction and heart failure.

**Conclusion:**

BSA, height, and BFP have more sensitivity in assessing cancer risk and BFP shows the largest hazard ratios for CVD incident. We provided valuable evidence for the application of height, BSA, and BFP in routine healthcare practice. These encouraging findings should be tested in more well‐defined studies for risk prediction.

## Graphical abstract

Body surface area, height, and body fat percentage has higher sensitivity in assessing cancer risk than BMI. Body fat percentage shows the largest hazard ratios in all cardiovascular events. BSA, height, and BFP have considerable application value in routine healthcare practice.

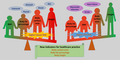

## 1 INTRODUCTION

Obesity and overweight defined by the body mass index (BMI) have been widely used and reported an increased risk of cancer or cardiovascular disease (CVD).^1^, ^2^, ^3^, ^4^, ^5^ However, BMI may not be the best biomarker to assess some diseases risk since the confusion of the “obesity paradox”,^6^, ^7^ that is, people with obesity usually showed more survival benefits such as lower mortality. Meanwhile, the BMI has certain limitations in distinguishing body fat and muscle component.^8^, ^9^ So many researchers strived to explore the potential value of other physical measurements.

Recently, some researchers explored the impact of height on various diseases,^5^, ^10^, ^11^, ^12^, ^13^ and found that tall stature was associated with increased risk for some specific cancer, but opposite for CVD.^5^, ^10^, ^11^, ^12^, ^13^ Besides, several studies have evaluated the effects of body fat percentage (BFP) and found a positive association with better survival (“obesity paradox”),^6^, ^14^, ^15^, ^16^, ^17^ however, evidence on the risk of incidence of cancer and cardiovascular disease remains limited. Moreover, studies have also reported evidence of a correlation between BSA and thyroid cancer and heart failure,^1^, ^18^ more extensive application values still deserve further exploration.

In the biological mechanism, height reflects early‐life factors like nutritional status in childhood and the genetic trait.^5^, ^10^ BMI and BFP are adiposity measures, representing the number and function of fat cells, metabolism of lipids, et al^19^, ^20^ Body surface area is highly correlated with basal metabolic rate,^21^ body composition,^22^ and commonly used as a hemodynamic parameter in clinical practice.^18^ These biological effects reflect by physical measurements may play important roles in the occurrence of cancer and CVD, which suggested the potential values of physical measures as biomarkers for some specific diseases. However, evidence for comparing the utility of these parameters on extensive diseases in one cohort is still limited.

We hypothesized that the effects of different physical measures on the risk of a particular disease in the same population are personalized. The objective of the study was to examine and compare the value of BMI, height, BSA, and BFP on cancer and cardiovascular disease as risk factors in guiding risk assessment in a large healthcare checkup population.

## 2 METHODS

### 2.1 Study participants

**Figure 1:**
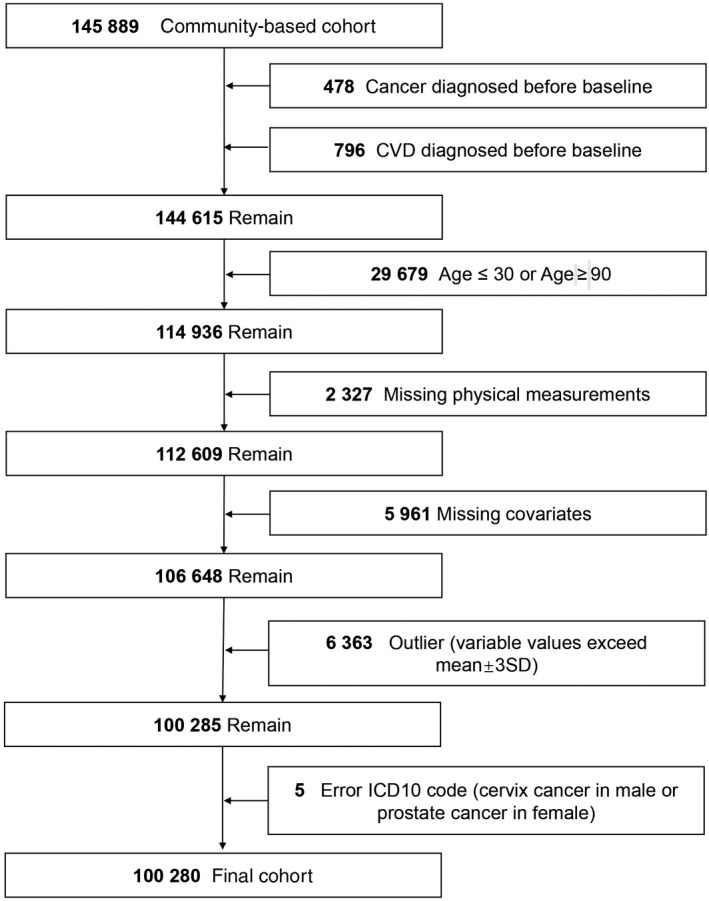
Flowchart of Inclusion and Exclusion Criteria for Participants

A community‐based cohort was created from the “Shandong Multi‐Center Healthcare Big Data Platform” (SMCHBDP) in China. A total of 145,889 subjects with a routine medical examination including physical measurement, blood biochemical examination, and disease diagnosis from May 2004 to December 2017 were enrolled. The follow‐up time started at the date of the first visit for an individual and ended at the date of incident cancer or CVD or the endpoint of December 2017. Samples that did not have a target outcome by the endpoint were defined as censored. We excluded the individuals who were diagnosed with cancer or CVD before baseline (n = 1274), aged less than 30 or more than 90 years old (n = 29 679), missing physical measurements (n = 2327), missing key covariates (n = 5961), outlier of variables (n = 6363), and some error ICD10 code in target outcomes (n = 5). The flowchart of eligible individuals is shown in Figure 1. The final sample size of the large cohort was 100,280 with the longest follow‐up exceeding 10 years and the mean follow‐up period was 5.57 years for overall cancer and 5.73 years for incident CVD.

All participants underwent annually examinations at designated health checkup centers or community healthcare centers. Medical results were collected and recorded by trained staff in multiple baseline assessment centers under standard procedures. The essential baseline information including age, gender, body mass index (BMI), systolic blood pressure (SBP), diastolic blood pressure (DBP), total cholesterol (TC), triglyceride (TG), high‐density lipoprotein cholesterol (HDL‐C), low‐density lipoprotein cholesterol (LDL‐C), and fasting blood glucose (FBG). Besides, some medical history such as diabetes mellitus, hypertension, chronic kidney disease, and cancer for each participant was also collected from these visiting centers and also linked with Electronic Medical Record (EMR) and Resident Medical Insurance Payment System (RMIPS) by Resident Identity Card Number (RIDN).

The data collection was conducted from 2017 to 2018 and the work was initiated by the Health Commission of Shandong Province and Shandong University. Researchers can only use the data on the SMCHBDP server with approval by the official review committee after encryption.

### 2.2 Body size measurements and outcome

All variables to be studied (BMI, BSA, and BFP) were calculated from height and weight as well as age‐ and gender‐based on specific algorithms. BMI was calculated by dividing weight in kilograms by the square of height in meters. BSA was calculated according to the Mosteller's formula [weight (kg) × height (cm)/3600]^1/2^, which has been widely adopted over the years and this formula generated values within 2% of others.^18^, ^23^, ^24^ It is recommended as an accurate measure to estimate BSA and is commonly used due to its simplicity and applicability in both clinical and laboratory medicine.^25^ Body fat percentage (BFP) was calculated using the equation: (1.20 × BMI) + (0.23 × age)− (10.8 × sex [1 for male and 0 for female])−5.40.^26^ These prediction formulas for BSA and BFP have been widely used and validated for effective physiological parameters.^1^, ^18^, ^27^

The outcome of this study was defined as the new development of primary cancer (overall and site‐specific cancer) and cardiovascular events (stroke, myocardial infarction, and heart failure) during the follow‐up period. Diagnostic results for medical history and cancer were recorded by both ICD‐10 code and disease name.

### 2.3 Statistical analysis

All continuous variables were summarized by mean ± standard deviation (SD) and compared using one‐way ANOVA analysis, whereas categorical variables by percentage and tested via Chi‐Squared statistics. Pearson correlation coefficient test and Cochran‐Armitage test were also applied for continuous and categorical variables, respectively, to explore the trend of baseline characteristics in body measurements. We further plotted cumulative incidence curves for each physical measurement subgroup by the Kaplan Meier (K‐M) method. The log‐rank test was used to compare the equality of survival curves across the subgroups. Proportional hazards assumptions were confirmed through the Schoenfeld residuals test. Cox proportional hazards regression model was used to estimate hazard ratios (HRs) and its 95% confidence intervals (CIs) for cancer and CVD. The predictive accuracy of Cox models was assessed by ten‐fold cross‐validation using C‐index and area under the receiver operating characteristic curve (AUC).

We assessed the associations between percentiles (tertiles) of physical measurements (height, BMI, BSA, and BFP) and the risk of cancer, CVD and specific subtypes. The HRs were presented by tertile values (T1, T2, and T3 represent low, middle, and high level) for sufficient sample size and number of cases in each subgroup and meaningful comparison between different measurements. Sex‐specific percentiles cut‐off points were conducted in our research because obvious natural difference exists in physical measurements across gender. The corresponding cut‐off criteria were as follows: height for male [T1 (≤170 cm), T2 (170‐174 cm), T3 (>174 cm)], height for female [T1 (≤158 cm), T2 (158‐163 cm), T3 (>163 cm)]; BMI for male [T1 (≤24.6 kg/m^2^), T2 (24.6‐27.3 kg/m^2^), T3 (>27.3 kg/m^2^)], BMI for female [T1 (≤22.5 kg/m^2^), T2 (22.5‐25.5 kg/m^2^), T3 (>25.5 kg/m^2^)]; BSA for male [T1 (≤1.84 m^2^), T2 (1.84‐1.98 m^2^), T3 (>1.98 m^2^)], BSA for female [T1 (≤1.6 m^2^), T2 (1.6‐1.71 m^2^), T3 (>1.71 m^2^)]; BFP for male [T1 (≤23.4%), T2 (23.4%‐27.4%), T3 (>27.4%)], BFP for female [T1 (≤31.3%), T2 (31.3%‐36.4%), T3 (>36.4%)].

In the multivariate Cox model, we adjusted the covariates including age, gender, HDL‐C, LDL‐C, TG, FBG, and SBP. These covariates were confirmed in the baseline description to be both relevant to physical measurements and as recognized risk factors for cancer or CVD, and therefore, they were considered as potential confounders to be adjusted. We used the middle interval (T2) as the reference (HR = 1.00) group as this interval is usually considered the most normal range. Using the middle group as a reference can help identify the risk of diseases in both higher (T3) or lower (T1) subgroup of body measurement, which could show some potential nonlinear relationship. We further take the three levels (T1, T2, and T3) as a continuous variable to detect the linear association by per level increased. The above processes were repeated and stratified by gender as subgroup analysis and excluded diabetes and CKD individuals as a sensitivity analysis. Also, we further categorized the body measurements by median (high vs. low) as a binary exposure to explore the robustness of our results in the trend of tertiles. Furthermore, we detected the differences in the HRs of the four physical measures for a specific outcome in all analyses by the Z‐test, which has been used elsewhere.^28^ All statistical analyses were carried out using the R software (Version 3.4.1) and *P* < .05 (two‐tailed) was considered statistically significant.

## 3 RESULTS

The baseline characteristics of the study population by tertile of physical measurements are presented in Table 1. Except for the TG in height subgroups and CKD in BSA subgroups, all recorded characteristics were significantly different (*P* < .001). Overall, BMI, BSA, and BFP were positively related to TC, TG, LDL‐C, SBP, DBP, FBG, as well as the incidence of hypertension, diabetes mellitus, and CKD. However, these results are exactly the opposite according to body height. Besides, participants were older in the lower BSA and height subgroup. The sex‐specific baseline characteristics and outcome during the follow‐up period are presented in Table S1. Of the 100,280 individuals, 3107 (3.10%) were diagnosed with cancer, 49% were female; 3721 (3.71%) had CVD and most of them were male (68%). Both age, HDL‐C, and BFP were significantly higher in females (*P* < .001), whereas the other characteristics were higher in males (*P* < .001).

**Table 1:** Baseline characteristics of study population by body measurement groups

Characteristic	Group of body measurements (Mean ± SD)^a^			*P* value
	Low (T1)^b^	Middle (T2)^b^	High (T3)^b^	
BMI				
Age, years	43.29 ± 11.15	45.71 ± 10.90	47.25 ± 11.40	<.001*, a
TC, mmol/L	4.70 ± 0.90	4.91 ± 0.93	5.04 ± 0.96	<.001*, a
TG, mmol/L	1.08 ± 0.64	1.42 ± 0.84	1.71 ± 0.92	<.001*, a
HDL‐C, mmol/L	1.49 ± 0.33	1.38 ± 0.31	1.31 ± 0.29	<.001*, a
LDL‐C, mmol/L	2.74 ± 0.63	2.92 ± 0.62	3.02 ± 0.61	<.001*, a
SBP, mm Hg	121.79 ± 16.63	128.13 ± 17.42	135.16 ± 17.92	<.001*, a
DBP, mm Hg	76.14 ± 11.59	80.21 ± 12.27	84.70 ± 12.62	<.001*, a
FBG, mmol/L	4.89 ± 0.65	5.07 ± 0.76	5.26 ± 0.87	<.001*, a
Body weight, kg	61.03 ± 8.47	70.21 ± 9.15	80.72 ± 11.71	<.001*, a
Hypertension, %	16.01	26.92	41.74	<.001*, a
Diabetes mellitus, %	1.61	3.39	5.75	<.001*, a
CKD, %	0.30	0.45	0.50	<.001*, a
Height				
Age, years	49.60 ± 12.47	44.57 ± 10.37	41.58 ± 8.94	<.001*, a
TC, mmol/L	4.97 ± 0.97	4.87 ± 0.93	4.79 ± 0.91	<.001*, a
TG, mmol/L	1.41 ± 0.82	1.41 ± 0.86	1.41 ± 0.87	.804
HDL‐C, mmol/L	1.41 ± 0.32	1.39 ± 0.32	1.38 ± 0.32	<.001*, a
LDL‐C, mmol/L	2.95 ± 0.62	2.88 ± 0.63	2.84 ± 0.63	<.001*, a
SBP, mm Hg	131.19 ± 19.46	127.30 ± 17.77	126.25 ± 16.60	<.001*, a
DBP, mm Hg	80.88 ± 12.84	80.15 ± 12.64	79.95 ± 12.46	<.001*, a
FBG, mmol/L	5.13 ± 0.83	5.06 ± 0.77	5.03 ± 0.73	<.001*, a
Body weight, kg	65.90 ± 10.65	70.60 ± 11.82	76.04 ± 13.64	<.001*, a
Hypertension, %	33.75	26.70	23.56	<.001*, a
Diabetes mellitus, %	4.44	3.43	2.77	<.001*, a
CKD, %	0.54	0.39	0.31	<.001*, a
BSA				
Age, years	46.35 ± 12.46	45.39 ± 10.93	44.50 ± 10.23	<.001*, a
TC, mmol/L	4.80 ± 0.94	4.89 ± 0.94	4.96 ± 0.94	<.001*, a
TG, mmol/L	1.16 ± 0.70	1.41 ± 0.83	1.65 ± 0.92	<.001*, a
HDL‐C, mmol/L	1.48 ± 0.33	1.38 ± 0.31	1.31 ± 0.29	<.001*, a
LDL‐C, mmol/L	2.81 ± 0.63	2.90 ± 0.63	2.96 ± 0.62	<.001*, a
SBP, mm Hg	124.87 ± 18.52	128.06 ± 17.82	132.14 ± 17.41	<.001*, a
DBP, mm Hg	77.38 ± 12.21	80.27 ± 12.43	83.39 ± 12.62	<.001*, a
FBG, mmol/L	4.96 ± 0.72	5.07 ± 0.78	5.19 ± 0.82	<.001*, a
Body weight, kg	59.95 ± 7.62	70.02 ± 7.96	81.99 ± 11.04	<.001*, a
Hypertension, %	22.10	27.20	35.35	<.001*, a
Diabetes mellitus, %	2.47	3.63	4.65	<.001*, a
CKD, %	0.43	0.37	0.45	.260
BFP				
Age, years	37.95 ± 6.65	44.64 ± 8.96	53.65 ± 11.50	<.001*, a
TC, mmol/L	4.61 ± 0.86	4.92 ± 0.92	5.12 ± 0.97	<.001*, a
TG, mmol/L	1.12 ± 0.69	1.44 ± 0.86	1.66 ± 0.89	<.001*, a
HDL‐C, mmol/L	1.46 ± 0.33	1.38 ± 0.32	1.33 ± 0.30	<.001*, a
LDL‐C, mmol/L	2.69 ± 0.62	2.92 ± 0.61	3.06 ± 0.60	<.001*, a
SBP, mm Hg	119.88 ± 14.64	127.28 ± 16.64	137.91 ± 18.30	<.001*, a
DBP, mm Hg	75.59 ± 11.14	80.40 ± 12.34	85.06 ± 12.63	<.001*, a
FBG, mmol/L	4.83 ± 0.58	5.05 ± 0.73	5.34 ± 0.90	<.001*, a
Body weight, kg	62.97 ± 9.73	70.83 ± 10.72	78.15 ± 12.73	<.001*, a
Hypertension, %	11.68	25.17	47.80	<.001*, a
Diabetes mellitus, %	0.84	2.75	7.15	<.001*, a
CKD, %	0.19	0.33	0.73	<.001*, a

Abbreviations: BFP, body fat percentage; BMI, body mass index; BSA, body surface area; CKD, Chronic kidney disease; CVD, cardiovascular disease; DBP, diastolic blood pressure; FBG, fasting blood glucose; HDL‐C, high‐density lipoprotein cholesterol; LDL‐C, low‐density lipoprotein cholesterol; SBP, systolic blood pressure; SD, standard deviation; TC, total cholesterol; TG, triglyceride.

^a^ Continuous variables were described by mean ± standard deviation (SD) and compared by one‐way ANOVA test; categorical variables were described by percentage (%) and compared via Chi‐Squared test.

^b^ The characteristics were presented by tertiles (T1, T2, T3) used sex‐specific percentiles cut‐off points, which means the low, middle, and high level for a body measurement (see method part).

* significant for trend test. Continuous variables were tested by Pearson correlation coefficient, categorical variables were used the Cochran‐Armitage test.

**Figure 2:**
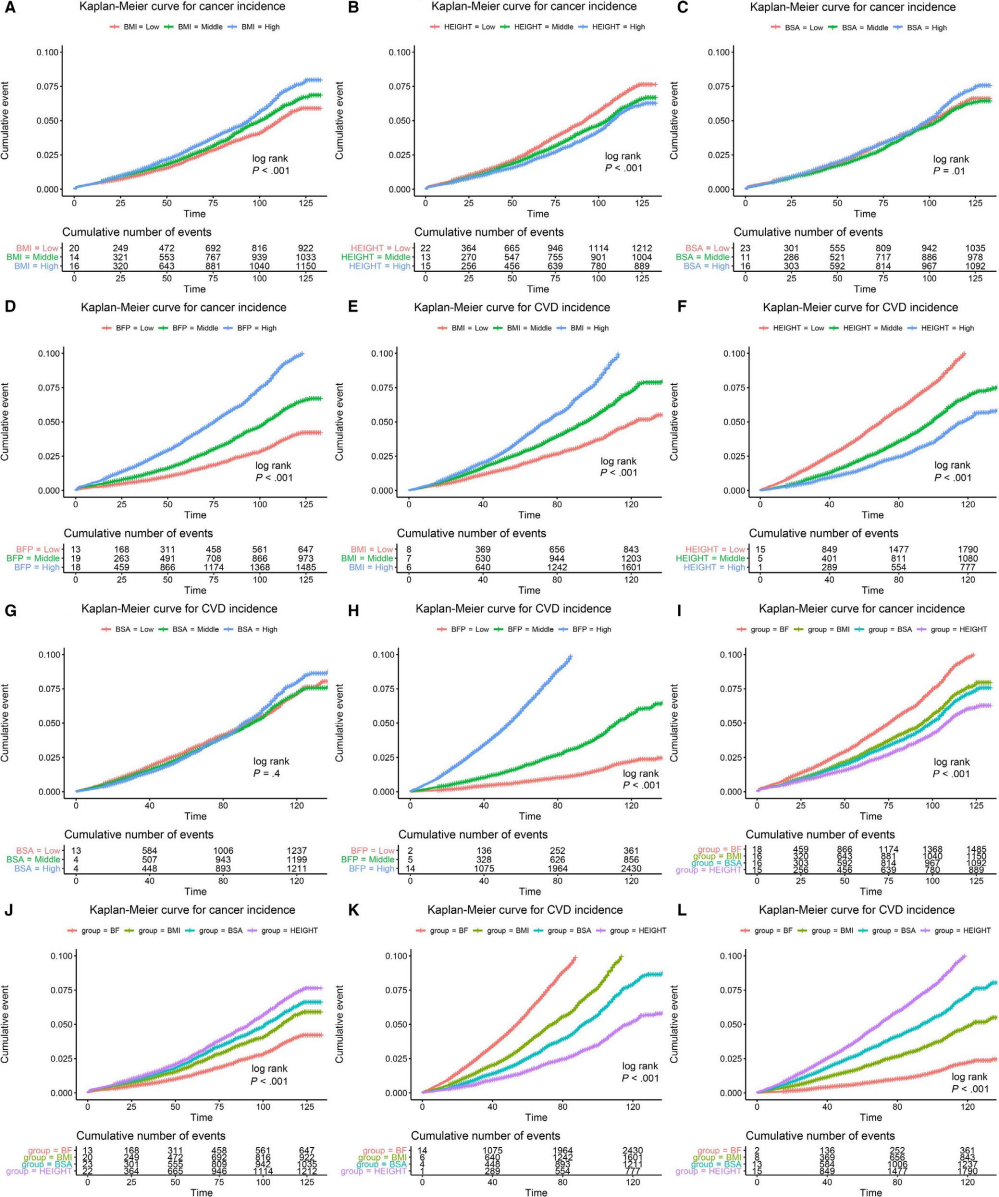
Cumulative Incidence Curve for Participants Based on Physical Measurement Subgroups. BMI, body mass index; BSA, body surface area; BFP, body fat percentage; CVD, Cardiovascular diseases. Each physical measurement is divided into three subgroups: low (T1), medium (T2) and high (T3). Part A‐D, showed the cumulative cancer incidence in the three subgroups of BMI, height, BSA, and BFP, respectively. Part E‐H, showed the cumulative CVD incidence in the three subgroups of BMI, height, BSA, and BFP, respectively. Part I and J showed the cumulative cancer incidence of the four physical measurements in the T1 and T3 group, respectively. Part K and L showed the cumulative CVD incidence of the four physical measurements in the T1 and T3 group, respectively

Figure 2 depicts the K‐M cumulative incidence curves for cancer and CVD events. BMI, height, and BFP subgroups were significantly different in cancer and CVD incidence (*P* < .001), whereas BSA was insignificant for CVD incidence (*P* = .4) (Figure 2A‐H). The incidence of cancer or CVD in the height subgroup was inversed to other measurements. The cumulative incidence curves of the four indicators were also significantly different in the T3 and T1 group (Figure 2I‐L). Schoenfeld test showed that all physical measurements satisfied the proportional hazards hypothesis (*P* > .05) except the BMI and BSA for CVD risk (Figure S1).

Table 2 showed the multivariate‐adjusted hazard ratios (HRs) and 95% confidence intervals (CIs) for the risk of cancer by tertiles of height, BMI, BSA, and BFP, respectively. Per level increased body height, BSA, and BFP were positively associated with the risk of overall cancer in all individuals except for BMI [HR (95% CI): 1.12 (1.07‐1.18), 1.10 (1.05‐1.15), and 1.10 (1.03‐1.16), respectively]. Compared with the T2 group, height and BFP in T1 group were significantly associated with decreased cancer incidence [HR (95% CI): 0.86 (0.79‐0.94) and 0.85 (0.76‐0.95)], whereas BSA in T3 group increased the cancer risk significantly [HR (95% CI): 1.19 (1.09‐1.30)]. The HR of BSA in the T3 group was significantly higher than the HRs of BMI and BFP (*P* < .05) when compared with T2 group, at the same time, the HRs of body height and BFP in the T1 group were also significantly different from the HRs of BMI and BSA (Table S2). For site‐specific cancer, BSA and height also displayed a strong positive association with thyroid cancer [HR (95%CI): 1.62 (1.27‐2.07) and 1.66 (1.29‐2.14), respectively] when comparing T3 to T2. All physical measurements increased the risk of thyroid and skin cancer in trend. Overall, the HRs of BSA and height were slightly higher for thyroid cancer, whereas the HRs of BSA and BFP were slightly higher for skin cancer. In addition, both lower (T1 level) and higher (T3 level) body height was protective for breast cancer when compared with T2, which showed an “inverted U‐shape” relationship. At the same time, lower (T1 level) BFP was also protectively for both breast cancer and colorectal cancer, but not significant in trend. In binary body measurements, higher (>median) height, BSA, and BDP still increased the cancer risk which inconsistent with the results in trend, meanwhile, we further found that height will increase the risk of urinary system cancer [HR (95%CI), 1.51 (1.06‐2.14)] (Table S3).

**Table 2:** Relationship between body measurements and cancer risk

Cancer Sites (No.)	BMI	Height	BSA	BFP
Overall cancer (3107)				
T1 (low)	0.97 (0.88‐1.06)	0.86 (0.79‐0.94)^b^, *	0.99 (0.91‐1.08)	0.85 (0.76‐0.95)^b^, *
T2 (reference)	1.00 (reference)	1.00 (reference)	1.00 (reference)	1.00 (reference)
T3 (high)	1.05 (0.97‐1.15)	1.09 (0.99‐1.19)	1.19 (1.09‐1.30)^b^, *	1.03 (0.94‐1.13)
HR for trend	1.04 (0.99‐1.09)	1.12 (1.07‐1.18)^b^, *	1.10 (1.05‐1.15)^b^, *	1.10 (1.03‐1.16)^b^, *
Lung (514)				
T1 (low)	1.12 (0.90‐1.40)	0.83 (0.68‐1.03)	1.04 (0.84‐1.29)	0.86 (0.66‐1.13)
T2 (reference)	1.00 (reference)	1.00 (reference)	1.00 (reference)	1.00 (reference)
T3 (high)	1.02 (0.82‐1.26)	0.95 (0.75‐1.20)	1.13 (0.91‐1.41)	0.84 (0.67‐1.05)
HR for trend	0.95 (0.85‐1.07)	1.08 (0.96‐1.21)	1.04 (0.93‐1.17)	0.96 (0.83‐1.12)
Breast (398)				
T1 (low)	0.83 (0.64‐1.07)	0.65 (0.51‐0.82)^b^, *	0.81 (0.63‐1.04)	0.72 (0.55‐0.95)^b^, *
T2 (reference)	1.00 (reference)	1.00 (reference)	1.00 (reference)	1.00 (reference)
T3 (high)	1.00 (0.79‐1.28)	0.77 (0.61‐0.99)^b^, *	0.95 (0.75‐1.20)	0.90 (0.69‐1.18)
HR for trend	1.10 (0.95‐1.26)	1.10 (0.96‐1.24)	1.08 (0.95‐1.23)	1.12 (0.94‐1.33)
Thyroid (366)				
T1 (low)	0.72 (0.54‐0.95)^b^, *	0.94 (0.72‐1.24)	0.86 (0.64‐1.15)	0.74 (0.55‐0.98)^b^, *
T2 (reference)	1.00 (reference)	1.00 (reference)	1.00 (reference)	1.00 (reference)
T3 (high)	1.14 (0.89‐1.46)	1.66 (1.29‐2.14)^b^, *	1.62 (1.27‐2.07)^b^, *	1.30 (0.99‐1.71)
HR for trend	1.25 (1.08‐1.44)^b^, *	1.35 (1.18‐1.54)^b^, *	1.40 (1.22‐1.60)^b^, *	1.33 (1.12‐1.57)^b^, *
Stomach (231)				
T1 (low)	0.88 (0.64‐1.23)	1.15 (0.83‐1.60)	0.92 (0.67‐1.26)	0.89 (0.59‐1.36)
T2 (reference)	1.00 (reference)	1.00 (reference)	1.00 (reference)	1.00 (reference)
T3 (high)	1.03 (0.75‐1.42)	1.47 (1.03‐2.10)^b^, *	1.17 (0.84‐1.64)	1.27 (0.90‐1.79)
HR for trend	1.08 (0.91‐1.28)	1.11 (0.93‐1.31)	1.13 (0.95‐1.34)	1.21 (0.96‐1.51)
Colorectal (219)				
T1 (low)	0.74 (0.53‐1.05)	0.86 (0.62‐1.19)	0.83 (0.60‐1.16)	0.54 (0.34‐0.85)^b^, *
T2 (reference)	1.00 (reference)	1.00 (reference)	1.00 (reference)	1.00 (reference)
T3 (high)	0.88 (0.64‐1.22)	1.14 (0.80‐1.62)	1.11 (0.79‐1.54)	0.92 (0.66‐1.29)
HR for trend	1.08 (0.90‐1.29)	1.15 (0.97‐1.37)	1.15 (0.97‐1.37)	1.21 (0.96‐1.53)
Liver (186)				
T1 (low)	1.15 (0.80‐1.65)	0.87 (0.62‐1.22)	1.30 (0.92‐1.85)	1.03 (0.67‐1.58)
T2 (reference)	1.00 (reference)	1.00 (reference)	1.00 (reference)	1.00 (reference)
T3 (high)	1.01 (0.70‐1.46)	0.95 (0.64‐1.41)	1.02 (0.68‐1.51)	0.73 (0.51‐1.07)
HR for trend	0.94 (0.77‐1.14)	1.06 (0.87‐1.28)	0.87 (0.72‐1.06)	0.83 (0.65‐1.06)
Lymphoma/Leukemia (139)				
T1 (low)	0.85 (0.55‐1.31)	0.76 (0.51‐1.12)	0.78 (0.52‐1.17)	1.00 (0.57‐1.75)
T2 (reference)	1.00 (reference)	1.00 (reference)	1.00 (reference)	1.00 (reference)
T3 (high)	0.95 (0.64‐1.41)	0.98 (0.63‐1.52)	0.93 (0.62‐1.40)	1.34 (0.86‐2.10)
HR for trend	1.05 (0.84‐1.31)	1.15 (0.92‐1.43)	1.09 (0.88‐1.36)	1.19 (0.89‐1.60)
Urinary system (141)				
T1 (low)	1.02 (0.66‐1.56)	0.86 (0.56‐1.30)	0.98 (0.64‐1.50)	0.90 (0.54‐1.48)
T2 (reference)	1.00 (reference)	1.00 (reference)	1.00 (reference)	1.00 (reference)
T3 (high)	1.11 (0.74‐1.67)	1.25 (0.82‐1.91)	1.28 (0.85‐1.94)	1.15 (0.74‐1.80)
HR for trend	1.05 (0.83‐1.31)	1.21 (0.97‐1.50)	1.15 (0.92‐1.43)	1.14 (0.86‐1.51)
Skin (111)				
T1 (low)	0.95 (0.56‐1.61)	0.81 (0.50‐1.32)	0.77 (0.45‐1.30)	0.75 (0.43‐1.30)
T2 (reference)	1.00 (reference)	1.00 (reference)	1.00 (reference)	1.00 (reference)
T3 (high)	1.84 (1.16‐2.92)^b^, *	1.45 (0.92‐2.28)	1.80 (1.15‐2.81)^b^, *	1.77 (1.09‐2.89)^b^, *
HR for trend	1.43 (1.10‐1.85)^b^, *	1.34 (1.05‐1.71)^b^, *	1.56 (1.22‐2.00)^b^, *	1.56 (1.14‐2.13)^b^, *
Cervix/Uterus (80)^*^, ^a^				
T1 (low)	0.94 (0.53‐1.67)	0.81 (0.47‐1.39)	0.90 (0.50‐1.60)	1.04 (0.57‐1.88)
T2 (reference)	1.00 (reference)	1.00 (reference)	1.00 (reference)	1.00 (reference)
T3 (high)	1.09 (0.63‐1.90)	0.96 (0.56‐1.66)	1.29 (0.76‐2.19)	0.96 (0.51‐1.81)
HR for trend	1.08 (0.79‐1.47)	1.09 (0.82‐1.44)	1.21 (0.91‐1.60)	0.96 (0.65‐1.42)
Prostate (58)^b^, ^*^				
T1 (low)	1.61 (0.83‐3.13)	1.29 (0.67‐2.47)	0.90 (0.49‐1.66)	0.64 (0.21‐1.96)
T2 (reference)	1.00 (reference)	1.00 (reference)	1.00 (reference)	1.00 (reference)
T3 (high)	1.30 (0.66‐2.58)	1.61 (0.73‐3.56)	0.97 (0.47‐1.97)	0.64 (0.34‐1.23)
HR for trend	0.89 (0.63‐1.25)	1.06 (0.74‐1.51)	1.05 (0.74‐1.48)	0.86 (0.53‐1.38)
Other sites (664)				
T1 (low)	1.00 (0.81‐1.22)	0.93 (0.78‐1.12)	1.11 (0.91‐1.34)	0.94 (0.75‐1.18)
T2 (reference)	1.00 (reference)	1.00 (reference)	1.00 (reference)	1.00 (reference)
T3 (high)	1.14 (0.95‐1.37)	1.01 (0.83‐1.23)	1.25 (1.03‐1.51)^b^, *	1.18 (0.96‐1.45)
HR for trend	1.07 (0.97‐1.19)	1.04 (0.94‐1.15)	1.06 (0.96‐1.17)	1.13 (0.99‐1.28)

Note

Abreviations; BMI, body mass index; BFP, body fat percentage; BSA, body surface area.

Continuous body measurements were divided into tertiles based on gender‐specific percentages, denoted as T1, T2, and T3, represented low, middle, and high level. T2 was set as the reference group with HR = 1.00. HR for trend represented the hazard ratio with per‐level body measurements increased. Relationship between body measurements and cancer was assessed by multivariate Cox model adjusted for age, sex, HDL‐C, LDL‐C, TG, FBG, SBP, and all results were reported by hazard ratios (HRs) and 95% confidence intervals (CIs).

^a^ only in female.

^b^ only in male.

* *P* < .05.

For males, BSA, height, and BFP also significantly increased the risk of overall cancer (HR = 1.13, 1.13 and 1.11 in trend, respectively, Table S4‐S5). Only BSA and height were significant in the T3 level when compared with T2 [HR (95%CI), 1.28 (1.13‐1.45) and 1.20 (1.05‐1.37)]. As for site‐specific cancer, except the thyroid [HR (95%CI), 1.72 (1.13‐2.62)] and skin cancer [HR (95%CI), 2.01 (1.05‐3.84)], BSA additional increased the risk of stomach cancer[HR (95%CI), 1.55 (1.03‐2.33)] and other sites [HR (95%CI), 1.33 (1.02‐1.73)] when compared T3 to T2, and also decreased the risk of Lymphoma/Leukemia [HR (95%CI), 0.63 (0.40‐1.00)] in T1. The BMI was insignificant with any cancer in the male. For females, all body measurements significantly increased the cancer risk in trend. When compared with the T2 level, BSA was positively associated with overall cancer in T3 [HR (95%CI), 1.13 (1.00‐1.28)], whereas height and BFP were significantly decreased the risk of cancer in T1 (Table S6‐S7). Higher BSA and height still increased the risk of thyroid cancer in the T3 level compared to T2 (HR = 1.59 and 1.90, respectively). The BMI also significantly associated with thyroid and skin cancer, whereas BFP and height additionally associated with breast cancer. The results were similar for binary body measurements (>Median vs. ≤Median) and the height additionally increased the risk of urinary system cancer for male [HR (95%CI), 1.68 (1.06‐2.65)] in Table S8.

Table 3 showed the relationship between physical measurements and the risk of CVD in the multivariate model. BFP, BMI, and BSA were significantly in relation to overall CVD risk in trend [HRs = 1.22, 1.09, 1.05, respectively]. Among these measurements, only BFP was significant in both the T1 group [HR (95%CI): 0.79 (0.70‐0.90)] and T3 group [HR (95%CI): 1.20 (1.10‐1.31)] and acquired the highest value at risk compared with others (Table S9). In CVD subtypes, higher BFP and BSA also increased the myocardial infarction (MI) risk by 44% [HR (95%CI): 1.44 (1.24‐1.67)] and 16%[HR (95%CI): 1.16 (1.01‐1.32)] when compared T3 to T2. All physical measurements increased the risk of heart failure (HF) in the trend test. As for stroke, only BFP presented a significant association in trend [HR (95%CI): 1.09 (1.01‐1.18)]. Overall, BFP had a positive correlation with nearly all CVD risks and showed the largest hazard ratios in the overall CVD and MI subtypes. Sensitivity analysis of binary body measurements still supports the above results (Table S10).

**Table 3:** Relationship between body measurements and risk of cardiovascular diseases

CVD (No.)	BMI	Height	BSA	BFP
Overall CVD (3721)				
T1	0.89 (0.82‐0.97)*	0.97 (0.90‐1.05)	0.96 (0.89‐1.05)	0.79 (0.70‐0.90)*
T2 (reference)	1.00 (reference)	1.00 (reference)	1.00 (reference)	1.00 (reference)
T3	1.07 (0.99‐1.15)	1.03 (0.94‐1.13)	1.07 (0.98‐1.15)	1.20 (1.10‐1.31)*
HR for trend	1.09 (1.05‐1.14)*	1.03 (0.99‐1.08)	1.05 (1.01‐1.10)*	1.22 (1.15‐1.30)*
Myocardial infarction (1251)				
T1	0.77 (0.66‐0.90)*	1.02 (0.89‐1.17)	1.01 (0.87‐1.16)	0.65 (0.52‐0.81)*
T2 (reference)	1.00 (reference)	1.00 (reference)	1.00 (reference)	1.00 (reference)
T3	1.07 (0.94‐1.22)	1.15 (0.99‐1.35)	1.16 (1.01‐1.32)*	1.44 (1.24‐1.67)*
HR for trend	1.16 (1.08‐1.26)*	1.05 (0.98‐1.14)	1.07 (1.00‐1.15)	1.47 (1.33‐1.64)*
Heart failure (603)				
T1	0.93 (0.75‐1.16)	0.82 (0.68‐0.99)*	0.81 (0.66‐0.99)*	0.78 (0.56‐1.10)
T2 (reference)	1.00 (reference)	1.00 (reference)	1.00 (reference)	1.00 (reference)
T3	1.24 (1.03‐1.50)*	1.05 (0.84‐1.32)	1.30 (1.07‐1.58)*	1.34 (1.07‐1.67)*
HR for trend	1.17 (1.05‐1.30)*	1.14 (1.03‐1.27)*	1.27 (1.15‐1.41)*	1.32 (1.13‐1.54)*
Stroke (2181)				
T1	0.95 (0.84‐1.06)	1.01 (0.91‐1.12)	1.04 (0.94‐1.15)	0.86 (0.73‐1.02)
T2 (reference)	1.00 (reference)	1.00 (reference)	1.00 (reference)	1.00 (reference)
T3	1.00 (0.91‐1.10)	0.99 (0.87‐1.12)	0.99 (0.89‐1.11)	1.05 (0.94‐1.18)
HR for trend	1.03 (0.97‐1.08)	0.99 (0.93‐1.05)	0.98 (0.92‐1.03)	1.09 (1.01‐1.18)*

Note

Abbreviations: BMI, body mass index; BFP, body fat percentage; BSA, body surface area; CVD, Cardiovascular diseases.

Continuous body measurements were divided into tertiles based on gender‐specific percentages, denoted as T1, T2, and T3 from low to high. T2 was set as the reference group. HR for trend represented the hazard ratio with per‐level body measurements increased. Relationship between body measurements and cancer was assessed by multivariate Cox model adjusted for age, sex, HDL‐C, LDL‐C, TG, FBG, SBP, and results were reported by hazard ratios (HRs) and 95% confidence intervals (CIs).

* *P* < .05.

Similar results were found in subgroup analysis by gender in Table S11. Only BFP and BMI were significantly in relation to overall CVD risk in both genders. The HRs and 95%CIs of per level increased BFP were 1.20 (1.12‐1.29) and 1.36 (1.20‐1.54) for males and females, respectively. As for MI, all physical measurements have a positive association in males but only BFP in the female. Besides, higher BFP, BSA, and height increased the HF risk in males, whereas higher BFP, BSA, and BMI increased the risk of HF in females for trend. BFP still showed the largest hazard ratios compared to other physical measurements in overall CVD and MI for both males and females (Table S12). Similar results also presented in binary body measurements (Table S13).

The results of interactions of physical measurements for overall cancer and CVD across gender are shown in Table 4. Among these physical measurements, only body height for cancer in the T3 group and BFP for CVD in trend was significantly different in gender subgroups (*P* for interaction <.05). Higher body height (T3 group) only increased the cancer risk in males when compared with T2 but not for females. In trend analysis, the HRs of height in males and females were consistent (*P* for interaction = .281). The risk of other physical measurements was consistent across gender. We also found there were no interactions between males and females in binary exposures which were consistent with the results in trend (Table S14). Sensitivity analysis excluded DM and CKD individuals (Table S15‐S19), previous results remained robust and did not change substantially. The predictive performance of Cox models for overall cancer and CVD was also assessed in Table S20. In univariate models, BFP has the largest AUC and C‐index for both cancer and CVD. After adjusted for potential confounders, the predictive performance was ideal and similar among four physical measurements for cancer (range of C‐index: 0.698‐0.700) and CVD (range of C‐index: 0.824‐0.825).

**Table 4:** Interactions of body measurements stratified by sex in overall cancer and cardiovascular diseases

Disease	Sex	Subgroup (T2 as a reference)	BMI		Height		BSA		BFP	
			HR (95%CI)	*P* for interaction	HR (95%CI)	*P* for interaction	HR (95%CI)	*P* for interaction	HR (95%CI)	*P* for interaction
Cancer	Male	T1 (low)	0.95 (0.84‐1.08)	0.333	0.93 (0.82‐1.05)	0.102	1.01 (0.89‐1.14)	0.174	0.87 (0.74‐1.01)	0.300
	Female	T1 (low)	0.92 (0.80‐1.05)		0.83 (0.73‐0.94)*		0.92 (0.81‐1.05)		0.82 (0.71‐0.95)*	
	Male	T3 (high)	1.07 (0.95‐1.21)	0.429	1.20 (1.05‐1.37)*	<0.05*	1.28 (1.13‐1.45)*	0.085	1.08 (0.95‐1.22)	0.298
	Female	T3 (high)	1.09 (0.96‐1.23)		1.01 (0.89‐1.14)		1.13 (1.00‐1.28)*		1.02 (0.89‐1.18)	
	Male	Trend	1.06 (0.99‐1.13)	0.287	1.13 (1.06‐1.21)*	0.281	1.13 (1.05‐1.20)*	0.377	1.11 (1.02‐1.20)*	0.463
	Female	Trend	1.09 (1.01‐1.17)*		1.10 (1.03‐1.18)*		1.11 (1.04‐1.18)*		1.11 (1.02‐1.22)*	
CVD	Male	T1 (low)	0.90 (0.81‐1.00)	0.102	0.97 (0.89‐1.07)	0.433	0.96 (0.87‐1.05)	0.421	0.82 (0.71‐0.95)*	0.092
	Female	T1 (low)	0.79 (0.66‐0.95)*		0.99 (0.86‐1.13)		0.94 (0.81‐1.09)		0.67 (0.52‐0.87)*	
	Male	T3 (high)	1.11 (1.01‐1.21)*	0.355	1.03 (0.92‐1.15)	0.482	1.09 (0.99‐1.21)	0.410	1.20 (1.08‐1.33)*	0.209
	Female	T3 (high)	1.07 (0.94‐1.22)		1.02 (0.86‐1.22)		1.07 (0.94‐1.23)		1.30 (1.10‐1.54)*	
	Male	Trend	1.11 (1.05‐1.17)*	0.248	1.03 (0.98‐1.08)	0.417	1.07 (1.02‐1.13)*	0.499	1.20 (1.12‐1.29)*	<0.05*
	Female	Trend	1.15 (1.05‐1.25)*		1.02 (0.94‐1.10)		1.07 (0.99‐1.15)		1.36 (1.20‐1.54)*	

Note

Abbreviations: BMI, body mass index; BFP, body fat percentage; BSA, body surface area; CVD, Cardiovascular diseases.

The difference of hazard ratios (HRs) in each group (interactions) were tested using two‐sample Z‐test.

Trend: per‐level increase among T1 (low), T2 (middle), and T3 (high).

* *P* < .05.

## 4 DISCUSSION

Our research has shown that BMI has a certain value for assessing the risk of some specific diseases, but it may not be the most suitable biological parameter. In comparison, BSA, height, and BFP displayed more exciting results for cancer and cardiovascular diseases. Since their clinical significance as risk factors were scantily investigated, the current results have made considerable contributions to the existing research progress, especially for guiding the healthcare in practice and the early risk assessment of some disease. First, both calculated on the basis of body height and weight, we found BSA and BFP as better physical examination index than BMI, particularly, which are simple and feasible equally in future applications. Second, we also revealed that an indicator has different sensitivities for different diseases, such as BSA, height, and BFP for cancer and BFP for cardiovascular and cerebrovascular events. This provides more evidence for selecting target biomarkers. Third, different physical measurements represent different biological meanings. Our results may contribute to the existing knowledge that will be useful for further research.

The results of this study were consistent with many reported findings. As for cancer risk assessment, however, body height, BMI, BSA, and BFP exhibited some differences. For instance, larger BSA was associated with a higher risk of thyroid for both males and females. Previous studies have also reported a dominant association between BSA and thyroid cancer since its important role in circulating blood volume, oxygen consumption, and basal energy expenditure.^29^ Study had shown that higher basal energy expenditure affecting serum thyroid stimulating hormone (TSH) peak levels,^30^ which may lead to an additional generation of TSH, a risk factor of thyroid cancer.^31^ Besides, increased thyroid cancer risk could be partially explained by a larger thyroid volume as BSA has been shown to be an independent predictor of the thyroid volume.^29^, ^32^ The larger number of thyroid epithelial cells among those with a higher BSA may increase the probability of a malignant transformation.^33^, ^34^ In addition, an increased risk of stomach and skin cancer with high‐level BSA was also found in our results for the male population. In our opinion, larger BSA means more skin cells and divisions, thus, increased the risk of skin cancer in a similar way as the above hypothesis.^35^ On the other hand, sun exposure especially the ultraviolet radiation in childhood may promote growth and development and therefore lead to greater BSA, however, it also accelerates the cutaneous DNA damage and potentially leading to mutagenesis and skin cancer.^36^, ^37^ This gender difference among some site‐specific cancers may be due to hormone dimorphism, and the natural differences in BSA and related metabolic consequences.^38^ Height, BMI, and BFP also reported associations with some cancer sites, including thyroid, stomach, skin, and breast cancer, but without stability across genders, and showed lower sensitivity as well. Another interesting finding is that, for some diseases, there is no excess risk in the higher group, but it was protective in the lower group, such as breast cancer. Although the exact mechanism is not clear, it still has important reference values in clinical practice.

Height also performed well in our results for cancer. Because height may be proportional to body surface area, it may have similar causes for the risk of thyroid and skin cancer. The excessive risk of stomach cancer in higher individuals may be attributable to the same way of a higher probability of a malignant transformation due to a larger stomach in tall stature. Another reason may be higher basal energy expenditure increased the requirement of energy and dietary and then increased the high‐calorie meals and more unhealthy ingredients, then burdened the digestive system. Besides, height also increased the risk of urinary system cancer in the male. One possible reason is that male's urinary tracts are more complex and longer than females. This potentially increases the harmful metabolites exposure in the urinary tract and increase the probability of carcinoma. Of course, there are still some limitations to the height that needs to be pointed out. First, higher body height presented lower cancer incident form from the cumulative incidence curve, it was rightly inversed with hazard ratios. This is not surprising, of course, because higher people tend to be younger. In developing China, with the improvement of lifestyle, the height of young is no longer limited by nutritional conditions, but the elder people may be limited when they grow. As a result, the effect of height may be more susceptible to the confounding of age. Second, the effect of height was heterogeneous across gender. Therefore, the results of height can be used as suggestive evidence, and its validity needs further verification.

In our analyses for CVD, the sensitivity of BSA has undergone an attenuation. Higher BSA only increased the risk of male's myocardial infarction and female's heart failure as well as the trend association for male's heart failure. Although lacking in epidemiological evidence of BSA and incident heart failure, a relevant study had demonstrated an inverse relationship between BSA and mortality in chronic HF patients.^18^ More evidence and potential mechanisms for their association require further research. In contrast to BSA, BMI performed well in most situations compared with the poor performance in cancer prediction, but still not the best one. It was worth paying attention to the BFP since it exhibited significant hazard ratios for nearly all of CVD and most were significant in trend except for male's stroke. Higher levels of BFP indicated an increased risk of 20% for overall CVD in male and 30% in the female. Besides, BFP also showed larger risk values for myocardial infarction and heart failure in both males and females. These results were consistent with several researches that noted higher percent trunk fat was associated with a particularly high risk of CVD^39^ and the visceral adiposity was associated with incident cardiovascular disease.^14^ It has been reported that relatively higher trunk fat levels were associated with various metabolic disturbances including worse glycemic control, elevated insulin levels, systemic inflammation, and dyslipidemia,^39^, ^40^, ^41^, ^42^, ^43^ which may accelerate the occurrence of CVD. Overall, BFP assesses CVD risk better than other indicators, and both sensitivity and risk values performed better. Therefore, adding BFP to a regular health checkup project is worth considering.

The advantage of our research is that we compared the prognostic values of several physical measurements for cancer and CVD in parallel for the first time. And we found that the commonly used BMI did not perform optimally in the risk assessment of them. Therefore, we provided critical evidence for the application of other physical measurements, including BSA and BFP. What's important is the ease of use of them just as BMI. Certainly, our research also has some limitations. For example, these indicators are based on calculation, which may have some differences compared to special instrument measurements. We also regret that we did have not obtained the data of waist to and hip ratio circumference as an additional comparison. Second, our research cannot address the estimation of disease risk in regional fat content. Third, we could not provide more detailed subtypes of CVD and mortality outcomes. Fourth, since our research was based on a large‐scale community, some information including tobacco, alcohol, and total energy intake was not collected. Finally, our sample size is still limited, and the results may still have some limitations for cancers with lower incidence. Therefore, we still need more research to verify our results.

In conclusion, our research compared the potential values of four physical measurements in cancer and CVD risk assessment. Overall, BSA, height, and BFP have more sensitivity in assessing cancer risk and BFP also shows the largest hazard ratios in measuring CVD risk. These indicated that physical measurements are personalized for disease risk. So this study provided evidence for the application of BSA, height, and BFP in routine clinical practice for their effectiveness and ease of use.

## CONFLICT OF INTEREST

The authors declare that they have no conflict of interest.

## AUTHOR CONTRIBUTIONS

All authors contributed to the study conception and design. Conception was proposed by Fuzhong Xue and Shucheng Si. Material preparation, data collection and analysis were performed by Shucheng Si. The first draft of the manuscript was written by Shucheng Si and all authors did the critical revision of the manuscript. Xiaokang Ji, Yongchao Wang, and Yanxun Liu contributed to acquire the research data. All authors commented on previous versions of the manuscript. All authors read and approved the final manuscript.

## ETHICS APPROVAL

All procedures involving human participants were performed in accordance with the ethical standards of the Ethics Committee of Public Health, Shandong University and the ethical standards as laid down in the 1964 Declaration of Helsinki and its later amendments or comparable ethical standards. (No.20190401).

## Supporting information

Supplementary Material

## Data Availability

Data cannot be shared publicly because these data are confidential and were obtained by signing a Data Use Agreement with the Health Commission of Shandong Province. The corresponding author Fuzhong Xue could help to submit an application to the official committee for researchers who meet the criteria for access to confidential data.
