# Supplementary material for: Body surface area, height, and body fat percentage as more sensitive risk factors of cancer and cardiovascular disease
Source: Cancer Med. 2020 Apr 27;9(12):4433–46. doi: 10.1002/cam4.3076 (PMC7300397; doi:10.1002/cam4.3076)
Supplement: Supplementary file 1 — Supplementary Material [file CAM4-9-4433-s001.docx]

**Body surface area, height, and body fat percentage as more sensitive risk factors of cancer and cardiovascular disease**

**CONTENT**

[Table S1. Baseline characteristic and outcome of study population by sex. 2](#_Toc35522669)

[Table S2. Difference (P value) in Hazard ratios among body measurements in cancer risk. 3](#_Toc35522670)

[Table S3. Relationship between body measurements and cancer risk group by median. 5](#_Toc35522671)

[Table S4. Relationship between body measurements and cancer risk in male. 6](#_Toc35522672)

[Table S5. Difference in Hazard ratios among body measurements in cancer risk for male. 8](#_Toc35522673)

[Table S6. Relationship between body measurements and cancer risk in female. 10](#_Toc35522674)

[Table S7. Difference in Hazard ratios among body measurements in cancer risk for female. 12](#_Toc35522675)

[Table S8. Relationship between body measurements and cancer risk in male and female group by median. 14](#_Toc35522676)

[Table S9. Difference in Hazard ratios among body measurements in CVD risk. 16](#_Toc35522677)

[Table S10. Relationship between body measurements and risk of CVD group by median. 17](#_Toc35522678)

[Table S11. Relationship between body measurements and risk of CVD in male and female. 18](#_Toc35522679)

[Table S12. Difference in hazard ratios among body measurements in CVD risk for male and female. 20](#_Toc35522680)

[Table S13. Relationship between body measurements and risk of CVD in male and female group by median. 21](#_Toc35522681)

[Table S15. Relationship between body measurements and cancer risk after excluded DM and CKD. 23](#_Toc35522682)

[Table S16. Relationship between body measurements and cancer risk in male after excluding DM and CKD. 25](#_Toc35522683)

[Table S17. Relationship between body measurements and cancer risk in female after excluding DM and CKD. 27](#_Toc35522684)

[Table S18. Relationship between body measurements and risk of CVD after excluding DM and CKD. 29](#_Toc35522685)

[Table S19. Relationship between body measurements and risk of CVD in male and female after excluding DM and CKD. 30](#_Toc35522686)

[Table20. Predictive performance of body measurements on overall cancer and CVD. 32](#_Toc35522687)

[Figure S1. Schoenfeld test of proportional hazards hypothesis for BMI, Height, BSA, and BFP. 33](#_Toc35522688)

Table S1. Baseline characteristic and outcome of study population by sex.

| **Characteristic** | **Total** | **Male** | **Female** | ***P* value** |
| --- | --- | --- | --- | --- |
| **Baseline** |  |  |  |  |
| No. | 100280 | 57666 | 42614 |  |
| Age, years | 45.41±11.27 | 45.30±11.34 | 45.56±11.18 | <0.001 |
| TC, mmol/L | 4.88±0.94 | 4.91±0.91 | 4.85±0.97 | <0.001 |
| TG, mmol/L | 1.41±0.85 | 1.58±0.91 | 1.17±0.67 | <0.001 |
| HDL-C, mmol/L | 1.39±0.32 | 1.32±0.30 | 1.49±0.31 | <0.001 |
| LDL-C, mmol/L | 2.89±0.63 | 2.94±0.63 | 2.83±0.63 | <0.001 |
| SBP, mmHg | 128.36±18.17 | 131.62±16.84 | 123.94±18.95 | <0.001 |
| DBP, mmHg | 80.35±12.66 | 83.90±12.16 | 75.54±11.70 | <0.001 |
| FBG, mmol/L | 5.07±0.78 | 5.15±0.82 | 4.97±0.71 | <0.001 |
| Height, cm | 166.85±8.28 | 171.74±6.15 | 160.23±5.81 | <0.001 |
| Body weight, kg | 70.65±12.74 | 76.81±11.46 | 62.30±9.11 | <0.001 |
| BMI, kg/m^2^ | 25.29±3.50 | 26.02±3.34 | 24.30±3.45 | <0.001 |
| BSA, m^2^ | 1.80±0.19 | 1.91±0.16 | 1.66±0.13 | <0.001 |
| BF, % | 29.18±6.73 | 25.44±4.77 | 34.24±5.59 | <0.001 |
| Hypertension, % | 28.21 | 34.26 | 20.03 | <0.001 |
| Diabetes mellitus, % | 3.58 | 4.20 | 2.75 | <0.001 |
| CKD, % | 0.42 | 0.53 | 0.27 | <0.001 |
| **Outcome** |  |  |  |  |
| No. of cancer, % | 3.10 | 2.75 | 3.57 | <0.001 |
| No. of CVD events, % | 3.71 | 4.41 | 2.77 | <0.001 |

Continuous variables were described by mean ± standard deviation (SD) and compared by t-test; categorical variables were described by N (%) and compared via Chi-Squared test.

Abbreviation: TC, total cholesterol; TG, triglyceride; HDL-C, high density lipoprotein cholesterol; LDL-C, low density lipoprotein cholesterol; SBP, systolic blood pressure; DBP, diastolic blood pressure; FBG, fasting blood glucose; BMI, body mass index; BSA, body surface area; BFP, body fat percentage; CKD, Chronic kidney disease; CVD, cardiovascular disease.

Table S2. Difference (P value) in Hazard ratios among body measurements in cancer risk.

| **Cancer Sites** | **HEIGHT vs. BMI** | **BSA vs. BMI** | **BFP vs. BMI** | **BSA vs. HEIGHT** | **BFP vs. HEIGHT** | **BSA vs. BFP** | |  |
| --- | --- | --- | --- | --- | --- | --- | --- | --- |
| **Overall cancer** |  |  |  |  |  | |  | |
| Low level (T1) | <0.05 | 0.38 | <0.05 | <0.05 | 0.41 | | <0.05 | |
| High level (T3) | 0.3 | <0.05 | 0.38 | 0.08 | 0.21 | | <0.05 | |
| Per level increased | <0.05 | 0.06 | 0.11 | 0.24 | 0.25 | | 0.47 | |
| **Lung** |  |  |  |  |  | |  | |
| Low level (T1) | <0.05 | 0.32 | 0.07 | 0.07 | 0.42 | | 0.15 | |
| High level (T3) | 0.34 | 0.25 | 0.11 | 0.14 | 0.23 | | <0.05 | |
| Per level increased | 0.07 | 0.14 | 0.46 | 0.34 | 0.12 | | 0.21 | |
| **Breast** |  |  |  |  |  | |  | |
| Low level (T1) | 0.09 | 0.44 | 0.24 | 0.11 | 0.28 | | 0.28 | |
| High level (T3) | 0.07 | 0.38 | 0.28 | 0.11 | 0.2 | | 0.38 | |
| Per level increased | 0.5 | 0.44 | 0.43 | 0.44 | 0.42 | | 0.38 | |
| **Thyroid** |  |  |  |  |  | |  | |
| Low level (T1) | 0.09 | 0.19 | 0.45 | 0.33 | 0.12 | | 0.23 | |
| High level (T3) | <0.05 | <0.05 | 0.24 | 0.44 | 0.1 | | 0.12 | |
| Per level increased | 0.22 | 0.13 | 0.29 | 0.35 | 0.44 | | 0.32 | |
| **Stomach** |  |  |  |  |  | |  | |
| Low level (T1) | 0.13 | 0.44 | 0.49 | 0.16 | 0.17 | | 0.46 | |
| High level (T3) | 0.07 | 0.29 | 0.19 | 0.19 | 0.28 | | 0.37 | |
| Per level increased | 0.41 | 0.35 | 0.22 | 0.43 | 0.28 | | 0.33 | |
| **Colorectal** |  |  |  |  |  | |  | |
| Low level (T1) | 0.27 | 0.32 | 0.14 | 0.45 | 0.05 | | 0.07 | |
| High level (T3) | 0.15 | 0.17 | 0.43 | 0.45 | 0.2 | | 0.22 | |
| Per level increased | 0.31 | 0.31 | 0.22 | 0.5 | 0.37 | | 0.37 | |
| **Liver** |  |  |  |  |  | |  | |
| Low level (T1) | 0.13 | 0.31 | 0.35 | 0.05 | 0.27 | | 0.2 | |
| High level (T3) | 0.41 | 0.49 | 0.12 | 0.41 | 0.17 | | 0.12 | |
| Per level increased | 0.19 | 0.3 | 0.23 | 0.08 | 0.07 | | 0.39 | |
| **Lymphoma/Leukemia** |  |  |  |  |  | |  | |
| Low level (T1) | 0.35 | 0.39 | 0.33 | 0.46 | 0.22 | | 0.25 | |
| High level (T3) | 0.46 | 0.47 | 0.13 | 0.44 | 0.16 | | 0.12 | |
| Per level increased | 0.29 | 0.41 | 0.25 | 0.37 | 0.42 | | 0.32 | |
| **Urinary system** |  |  |  |  |  | |  | |
| Low level (T1) | 0.29 | 0.45 | 0.35 | 0.33 | 0.45 | | 0.39 | |
| High level (T3) | 0.34 | 0.31 | 0.45 | 0.47 | 0.4 | | 0.37 | |
| Per level increased | 0.18 | 0.28 | 0.33 | 0.37 | 0.37 | | 0.48 | |
| **Skin** |  |  |  |  |  | |  | |
| Low level (T1) | 0.33 | 0.28 | 0.27 | 0.44 | 0.41 | | 0.48 | |
| High level (T3) | 0.23 | 0.47 | 0.45 | 0.25 | 0.28 | | 0.48 | |
| Per level increased | 0.36 | 0.32 | 0.34 | 0.2 | 0.23 | | 0.5 | |
| **Cervix/Uterus** |  |  |  |  |  | |  | |
| Low level (T1) | 0.36 | 0.46 | 0.41 | 0.4 | 0.27 | | 0.37 | |
| High level (T3) | 0.37 | 0.33 | 0.38 | 0.22 | 0.5 | | 0.24 | |
| Per level increased | 0.48 | 0.3 | 0.32 | 0.31 | 0.31 | | 0.18 | |
| **Prostate** |  |  |  |  |  | |  | |
| Low level (T1) | 0.32 | 0.1 | 0.08 | 0.21 | 0.15 | | 0.3 | |
| High level (T3) | 0.35 | 0.28 | 0.07 | 0.17 | <0.05 | | 0.2 | |
| Per level increased | 0.25 | 0.26 | 0.45 | 0.48 | 0.25 | | 0.26 | |
| **Other sites** |  |  |  |  |  | |  | |
| Low level (T1) | 0.32 | 0.23 | 0.36 | 0.11 | 0.48 | | 0.14 | |
| High level (T3) | 0.19 | 0.26 | 0.4 | 0.07 | 0.14 | | 0.36 | |
| Per level increased | 0.35 | 0.44 | 0.28 | 0.4 | 0.18 | | 0.24 | |

The difference of hazard ratios (HRs) in each group were tested using two-sample Z-test.

Table S3. Relationship between body measurements and cancer risk group by median.

| **Cancer Sites** | **BMI** | **Height** | **BSA** | **BFP** |
| --- | --- | --- | --- | --- |
| **Overall cancer** |  |  |  |  |
| ≤Median | 1.00(reference) | 1.00(reference) | 1.00(reference) | 1.00(reference) |
| >Median | 1.06(0.98-1.15) | 1.18(1.09-1.27)* | 1.14(1.06-1.23)* | 1.13(1.03-1.24)* |
| **Lung** |  |  |  |  |
| ≤Median | 1.00(reference) | 1.00(reference) | 1.00(reference) | 1.00(reference) |
| >Median | 0.88(0.73-1.06) | 1.08(0.90-1.30) | 1.04(0.87-1.25) | 0.98(0.78-1.24) |
| **Breast** |  |  |  |  |
| ≤Median | 1.00(reference) | 1.00(reference) | 1.00(reference) | 1.00(reference) |
| >Median | 1.10(0.88-1.37) | 1.10(0.89-1.35) | 1.23(1.00-1.51) | 1.12(0.86-1.46) |
| **Thyroid** |  |  |  |  |
| ≤Median | 1.00(reference) | 1.00(reference) | 1.00(reference) | 1.00(reference) |
| >Median | 1.51(1.19-1.90)* | 1.48(1.19-1.83)* | 1.42(1.14-1.76)* | 1.40(1.08-1.81)* |
| **Stomach** |  |  |  |  |
| ≤Median | 1.00(reference) | 1.00(reference) | 1.00(reference) | 1.00(reference) |
| >Median | 1.18(0.89-1.56) | 1.21(0.92-1.59) | 1.16(0.88-1.53) | 1.35(0.95-1.91) |
| **Colorectal** |  |  |  |  |
| ≤Median | 1.00(reference) | 1.00(reference) | 1.00(reference) | 1.00(reference) |
| >Median | 1.08(0.81-1.44) | 1.18(0.89-1.56) | 0.99(0.75-1.31) | 1.42(0.99-2.05) |
| **Liver** |  |  |  |  |
| ≤Median | 1.00(reference) | 1.00(reference) | 1.00(reference) | 1.00(reference) |
| >Median | 1.05(0.77-1.43) | 1.00(0.73-1.36) | 0.79(0.58-1.08) | 0.85(0.58-1.24) |
| **Lymphoma/Leukemia** |  |  |  |  |
| ≤Median | 1.00(reference) | 1.00(reference) | 1.00(reference) | 1.00(reference) |
| >Median | 1.09(0.76-1.56) | 1.18(0.83-1.68) | 1.16(0.82-1.65) | 0.98(0.63-1.54) |
| **Urinary system** |  |  |  |  |
| ≤Median | 1.00(reference) | 1.00(reference) | 1.00(reference) | 1.00(reference) |
| >Median | 1.02(0.71-1.47) | 1.51(1.06-2.14)* | 1.30(0.91-1.84) | 1.00(0.65-1.56) |
| **Skin** |  |  |  |  |
| ≤Median | 1.00(reference) | 1.00(reference) | 1.00(reference) | 1.00(reference) |
| >Median | 1.70(1.12-2.58)* | 1.54(1.04-2.28)* | 2.30(1.52-3.48)* | 1.73(1.08-2.79)* |
| **Cervix/Uterus** |  |  |  |  |
| ≤Median | 1.00(reference) | 1.00(reference) | 1.00(reference) | 1.00(reference) |
| >Median | 0.90(0.55-1.47) | 1.26(0.79-1.99) | 1.55(0.97-2.47) | 0.86(0.48-1.56) |
| **Prostate** |  |  |  |  |
| ≤Median | 1.00(reference) | 1.00(reference) | 1.00(reference) | 1.00(reference) |
| >Median | 0.96(0.56-1.66) | 1.39(0.79-2.43) | 0.74(0.41-1.33) | 0.63(0.31-1.31) |
| **Other sites** |  |  |  |  |
| ≤Median | 1.00(reference) | 1.00(reference) | 1.00(reference) | 1.00(reference) |
| >Median | 1.09(0.93-1.29) | 1.08(0.92-1.27) | 1.16(0.99-1.36) | 1.26(1.03-1.53)* |

Abbreviation: BMI, body mass index; BSA, body surface area; BFP, body fat percentage.

Table S4. Relationship between body measurements and cancer risk in male.

| **Cancer Sites** | **BMI** | **Height** | **BSA** | **BFP** |
| --- | --- | --- | --- | --- |
| **Overall cancer** |  |  |  |  |
| T1 | 0.95(0.84-1.08) | 0.93(0.82-1.05) | 1.01(0.89-1.14) | 0.87(0.74-1.01) |
| T2(ref group) | 1.00(reference) | 1.00(reference) | 1.00(reference) | 1.00(reference) |
| T3 | 1.07(0.95-1.21) | 1.20(1.05-1.37)* | 1.28(1.13-1.45)* | 1.08(0.95-1.22) |
| HR for trend | 1.06(0.99-1.13) | 1.13(1.06-1.21)* | 1.13(1.05-1.20)* | 1.11(1.02-1.20)* |
| **Lung** |  |  |  |  |
| T1 | 1.30(0.98-1.72) | 0.88(0.68-1.15) | 1.07(0.82-1.40) | 1.00(0.71-1.40) |
| T2(ref group) | 1.00(reference) | 1.00(reference) | 1.00(reference) | 1.00(reference) |
| T3 | 1.14(0.85-1.52) | 1.01(0.75-1.37) | 1.06(0.78-1.42) | 0.85(0.64-1.12) |
| HR for trend | 0.93(0.80-1.08) | 1.07(0.93-1.24) | 0.99(0.85-1.15) | 0.91(0.76-1.10) |
| **Breast** |  |  |  |  |
| T1 | 0.35(0.10-1.17) | 0.36(0.12-1.07) | 1.29(0.37-4.55) | 0.23(0.05-1.20) |
| T2(ref group) | 1.00(reference) | 1.00(reference) | 1.00(reference) | 1.00(reference) |
| T3 | 0.70(0.23-2.13) | 0.34(0.09-1.25) | 2.32(0.67-8.06) | 1.35(0.45-3.98) |
| HR for trend | 1.37(0.74-2.55) | 1.06(0.58-1.93) | 1.35(0.73-2.49) | 2.07(0.97-4.42) |
| **Thyroid** |  |  |  |  |
| T1 | 0.62(0.37-1.04) | 0.70(0.43-1.13) | 0.66(0.38-1.14) | 0.91(0.54-1.52) |
| T2(ref group) | 1.00(reference) | 1.00(reference) | 1.00(reference) | 1.00(reference) |
| T3 | 1.07(0.71-1.61) | 1.30(0.85-1.98) | 1.72(1.13-2.62)* | 1.44(0.92-2.27) |
| HR for trend | 1.27(0.99-1.63) | 1.36(1.08-1.71)* | 1.63(1.27-2.10)* | 1.28(0.97-1.69) |
| **Stomach** |  |  |  |  |
| T1 | 0.78(0.53-1.14) | 1.17(0.79-1.71) | 1.02(0.69-1.50) | 0.74(0.43-1.26) |
| T2(ref group) | 1.00(reference) | 1.00(reference) | 1.00(reference) | 1.00(reference) |
| T3 | 1.11(0.76-1.62) | 1.40(0.91-2.17) | 1.55(1.03-2.33)* | 1.33(0.90-1.97) |
| HR for trend | 1.19(0.97-1.47) | 1.07(0.88-1.31) | 1.22(1.00-1.50) | 1.34(1.03-1.75)* |
| **Colorectal** |  |  |  |  |
| T1 | 0.77(0.52-1.16) | 0.81(0.55-1.19) | 0.89(0.60-1.32) | 0.51(0.29-0.90)* |
| T2(ref group) | 1.00(reference) | 1.00(reference) | 1.00(reference) | 1.00(reference) |
| T3 | 0.95(0.65-1.39) | 1.19(0.78-1.80) | 1.27(0.85-1.90) | 0.93(0.63-1.36) |
| HR for trend | 1.10(0.89-1.36) | 1.21(0.99-1.49) | 1.20(0.97-1.48) | 1.22(0.93-1.60) |
| **Liver** |  |  |  |  |
| T1 | 1.05(0.71-1.56) | 0.91(0.62-1.34) | 1.20(0.82-1.76) | 0.86(0.53-1.37) |
| T2(ref group) | 1.00(reference) | 1.00(reference) | 1.00(reference) | 1.00(reference) |
| T3 | 0.91(0.60-1.38) | 1.03(0.67-1.59) | 0.95(0.61-1.50) | 0.66(0.44-0.99)* |
| HR for trend | 0.93(0.75-1.15) | 1.07(0.86-1.32) | 0.88(0.71-1.10) | 0.85(0.66-1.11) |
| **Lymphoma/Leukemia** |  |  |  |  |
| T1 | 0.76(0.47-1.24) | 0.67(0.43-1.05) | 0.63(0.40-1.00)* | 0.92(0.48-1.75) |
| T2(ref group) | 1.00(reference) | 1.00(reference) | 1.00(reference) | 1.00(reference) |
| T3 | 0.96(0.61-1.50) | 0.90(0.55-1.47) | 0.88(0.55-1.40) | 1.33(0.81-2.18) |
| HR for trend | 1.11(0.86-1.43) | 1.17(0.92-1.50) | 1.18(0.92-1.52) | 1.23(0.89-1.71) |
| **Urinary system** |  |  |  |  |
| T1 | 0.78(0.44-1.38) | 1.01(0.58-1.77) | 0.95(0.54-1.67) | 0.74(0.38-1.46) |
| T2(ref group) | 1.00(reference) | 1.00(reference) | 1.00(reference) | 1.00(reference) |
| T3 | 1.28(0.76-2.17) | 1.49(0.85-2.63) | 1.49(0.86-2.58) | 1.27(0.73-2.21) |
| HR for trend | 1.29(0.96-1.73) | 1.21(0.92-1.61) | 1.25(0.94-1.67) | 1.30(0.91-1.86) |
| **Skin** |  |  |  |  |
| T1 | 0.95(0.48-1.89) | 1.03(0.50-2.12) | 1.00(0.49-2.04) | 0.76(0.37-1.60) |
| T2(ref group) | 1.00(reference) | 1.00(reference) | 1.00(reference) | 1.00(reference) |
| T3 | 1.52(0.81-2.89) | 2.02(1.06-3.88)* | 2.01(1.05-3.84)* | 1.70(0.88-3.28) |
| HR for trend | 1.28(0.90-1.81) | 1.44(1.03-2.01)* | 1.45(1.03-2.05)* | 1.51(1.01-2.26)* |
| **Prostate** |  |  |  |  |
| T1 | 1.61(0.83-3.13) | 1.29(0.67-2.47) | 0.90(0.49-1.66) | 0.64(0.21-1.96) |
| T2(ref group) | 1.00(reference) | 1.00(reference) | 1.00(reference) | 1.00(reference) |
| T3 | 1.30(0.66-2.58) | 1.61(0.73-3.56) | 0.97(0.47-1.97) | 0.64(0.34-1.23) |
| HR for trend | 0.89(0.63-1.25) | 1.06(0.74-1.51) | 1.05(0.74-1.48) | 0.86(0.53-1.38) |
| **Other sites** |  |  |  |  |
| T1 | 0.94(0.72-1.22) | 1.05(0.81-1.35) | 1.09(0.84-1.42) | 1.04(0.76-1.43) |
| T2(ref group) | 1.00(reference) | 1.00(reference) | 1.00(reference) | 1.00(reference) |
| T3 | 1.05(0.81-1.35) | 1.29(0.98-1.69) | 1.33(1.02-1.73)* | 1.32(1.01-1.73)* |
| HR for trend | 1.06(0.92-1.22) | 1.10(0.96-1.26) | 1.10(0.96-1.26) | 1.15(0.97-1.36) |

Abbreviation: BMI, body mass index; BSA, body surface area; BFP, body fat percentage.

Continuous body measurements were divided into tertiles based on gender-specific percentages, denoted as T1, T2, and T3 from low to high. T2 was set as the reference group.

* P<0.05.

Table S5. Difference in Hazard ratios among body measurements in cancer risk for male.

| **Cancer Sites** | **HEIGHT vs. BMI** | **BSA vs. BMI** | **BFP vs. BMI** | **BSA vs. HEIGHT** | **BFP vs. HEIGHT** | **BSA vs. BFP** |
| --- | --- | --- | --- | --- | --- | --- |
| **Overall cancer** |  |  |  |  |  |  |
| Low level (T1) | 0.37 | 0.28 | 0.17 | 0.18 | 0.25 | 0.07 |
| High level (T3) | 0.1 | <0.05 | 0.47 | 0.25 | 0.12 | <0.05 |
| Per level increased | 0.07 | 0.1 | 0.2 | 0.43 | 0.33 | 0.39 |
| **Lung** |  |  |  |  |  |  |
| Low level (T1) | <0.05 | 0.16 | 0.12 | 0.16 | 0.3 | 0.37 |
| High level (T3) | 0.29 | 0.36 | 0.08 | 0.42 | 0.2 | 0.15 |
| Per level increased | 0.09 | 0.29 | 0.43 | 0.22 | 0.09 | 0.25 |
| **Breast** |  |  |  |  |  |  |
| Low level (T1) | 0.48 | 0.07 | 0.35 | 0.07 | 0.34 | 0.05 |
| High level (T3) | 0.2 | 0.08 | 0.2 | <0.05 | 0.06 | 0.26 |
| Per level increased | 0.28 | 0.49 | 0.2 | 0.29 | 0.09 | 0.19 |
| **Thyroid** |  |  |  |  |  |  |
| Low level (T1) | 0.38 | 0.44 | 0.16 | 0.44 | 0.23 | 0.2 |
| High level (T3) | 0.26 | 0.06 | 0.17 | 0.18 | 0.37 | 0.29 |
| Per level increased | 0.35 | 0.08 | 0.49 | 0.15 | 0.37 | 0.1 |
| **Stomach** |  |  |  |  |  |  |
| Low level (T1) | 0.07 | 0.17 | 0.44 | 0.31 | 0.09 | 0.17 |
| High level (T3) | 0.21 | 0.12 | 0.26 | 0.37 | 0.43 | 0.3 |
| Per level increased | 0.23 | 0.43 | 0.25 | 0.18 | 0.1 | 0.3 |
| **Colorectal** |  |  |  |  |  |  |
| Low level (T1) | 0.44 | 0.32 | 0.12 | 0.37 | 0.09 | 0.06 |
| High level (T3) | 0.22 | 0.15 | 0.47 | 0.41 | 0.2 | 0.13 |
| Per level increased | 0.26 | 0.29 | 0.28 | 0.47 | 0.48 | 0.45 |
| **Liver** |  |  |  |  |  |  |
| Low level (T1) | 0.3 | 0.32 | 0.25 | 0.16 | 0.42 | 0.14 |
| High level (T3) | 0.34 | 0.44 | 0.14 | 0.41 | 0.07 | 0.12 |
| Per level increased | 0.19 | 0.38 | 0.31 | 0.11 | 0.1 | 0.42 |
| **Lymphoma/Leukemia** |  |  |  |  |  |  |
| Low level (T1) | 0.36 | 0.29 | 0.33 | 0.42 | 0.22 | 0.18 |
| High level (T3) | 0.42 | 0.4 | 0.17 | 0.48 | 0.13 | 0.11 |
| Per level increased | 0.38 | 0.36 | 0.31 | 0.48 | 0.41 | 0.43 |
| **Urinary system** |  |  |  |  |  |  |
| Low level (T1) | 0.26 | 0.31 | 0.46 | 0.44 | 0.25 | 0.29 |
| High level (T3) | 0.35 | 0.35 | 0.49 | 0.5 | 0.34 | 0.34 |
| Per level increased | 0.39 | 0.45 | 0.48 | 0.44 | 0.39 | 0.44 |
| **Skin** |  |  |  |  |  |  |
| Low level (T1) | 0.44 | 0.46 | 0.33 | 0.48 | 0.28 | 0.3 |
| High level (T3) | 0.27 | 0.27 | 0.41 | 0.5 | 0.36 | 0.36 |
| Per level increased | 0.31 | 0.3 | 0.27 | 0.48 | 0.43 | 0.45 |
| **Prostate** |  |  |  |  |  |  |
| Low level (T1) | 0.32 | 0.1 | 0.08 | 0.21 | 0.15 | 0.3 |
| High level (T3) | 0.35 | 0.28 | 0.07 | 0.17 | <0.05 | 0.2 |
| Per level increased | 0.25 | 0.26 | 0.45 | 0.48 | 0.25 | 0.26 |
| **Other sites** |  |  |  |  |  |  |
| Low level (T1) | 0.28 | 0.21 | 0.31 | 0.41 | 0.49 | 0.41 |
| High level (T3) | 0.14 | 0.11 | 0.11 | 0.44 | 0.45 | 0.49 |
| Per level increased | 0.34 | 0.35 | 0.23 | 0.48 | 0.35 | 0.34 |

The difference of hazard ratios (HRs) in each group were tested using two-sample Z-test.

Table S6. Relationship between body measurements and cancer risk in female.

| **Cancer Sites (No.)** | **BMI** | **Height** | **BSA** | **BFP** |
| --- | --- | --- | --- | --- |
| **Overall cancer** |  |  |  |  |
| T1 | 0.92(0.80-1.05) | 0.83(0.73-0.94)* | 0.92(0.81-1.05) | 0.82(0.71-0.95)* |
| T2(ref group) | 1.00(reference) | 1.00(reference) | 1.00(reference) | 1.00(reference) |
| T3 | 1.09(0.96-1.23) | 1.01(0.89-1.14) | 1.13(1.00-1.28)* | 1.02(0.89-1.18) |
| HR for trend | 1.09(1.01-1.17)* | 1.10(1.03-1.18)* | 1.11(1.04-1.18)* | 1.11(1.02-1.22)* |
| **Lung** |  |  |  |  |
| T1 | 0.81(0.56-1.19) | 0.77(0.56-1.08) | 0.95(0.67-1.37) | 0.66(0.42-1.04) |
| T2(ref group) | 1.00(reference) | 1.00(reference) | 1.00(reference) | 1.00(reference) |
| T3 | 0.88(0.63-1.23) | 0.87(0.59-1.26) | 1.22(0.87-1.71) | 0.86(0.59-1.26) |
| HR for trend | 1.02(0.84-1.25) | 1.08(0.89-1.30) | 1.13(0.95-1.35) | 1.10(0.85-1.43) |
| **Breast** |  |  |  |  |
| T1 | 0.85(0.65-1.11) | 0.67(0.52-0.85)* | 0.78(0.60-1.01) | 0.75(0.57-0.99)* |
| T2(ref group) | 1.00(reference) | 1.00(reference) | 1.00(reference) | 1.00(reference) |
| T3 | 1.03(0.80-1.32) | 0.80(0.63-1.03) | 0.92(0.72-1.17) | 0.88(0.67-1.17) |
| HR for trend | 1.09(0.95-1.26) | 1.10(0.96-1.25) | 1.08(0.95-1.23) | 1.09(0.91-1.30) |
| **Thyroid** |  |  |  |  |
| T1 | 0.77(0.55-1.08) | 1.10(0.79-1.55) | 0.95(0.67-1.34) | 0.68(0.48-0.97)* |
| T2(ref group) | 1.00(reference) | 1.00(reference) | 1.00(reference) | 1.00(reference) |
| T3 | 1.20(0.89-1.63) | 1.90(1.38-2.60)* | 1.59(1.17-2.15)* | 1.26(0.89-1.77) |
| HR for trend | 1.25(1.04-1.49)* | 1.34(1.14-1.57)* | 1.32(1.12-1.55)* | 1.36(1.09-1.69)* |
| **Stomach** |  |  |  |  |
| T1 | 1.14(0.62-2.10) | 1.18(0.63-2.20) | 0.70(0.39-1.26) | 1.20(0.59-2.43) |
| T2(ref group) | 1.00(reference) | 1.00(reference) | 1.00(reference) | 1.00(reference) |
| T3 | 0.92(0.50-1.69) | 1.64(0.87-3.10) | 0.68(0.38-1.23) | 1.13(0.56-2.30) |
| HR for trend | 0.90(0.64-1.26) | 1.17(0.85-1.61) | 0.99(0.73-1.34) | 0.97(0.62-1.52) |
| **Colorectal** |  |  |  |  |
| T1 | 0.65(0.34-1.28) | 1.05(0.58-1.91) | 0.71(0.39-1.31) | 0.60(0.27-1.34) |
| T2(ref group) | 1.00(reference) | 1.00(reference) | 1.00(reference) | 1.00(reference) |
| T3 | 0.80(0.45-1.42) | 1.04(0.53-2.06) | 0.84(0.47-1.51) | 0.93(0.48-1.80) |
| HR for trend | 1.07(0.76-1.51) | 0.99(0.71-1.38) | 1.08(0.79-1.47) | 1.19(0.75-1.89) |
| **Liver** |  |  |  |  |
| T1 | 1.57(0.62-3.96) | 0.70(0.33-1.49) | 1.81(0.79-4.18) | 2.23(0.77-6.49) |
| T2(ref group) | 1.00(reference) | 1.00(reference) | 1.00(reference) | 1.00(reference) |
| T3 | 1.41(0.62-3.23) | 0.70(0.28-1.74) | 1.27(0.54-3.00) | 1.25(0.46-3.42) |
| HR for trend | 0.99(0.62-1.56) | 1.04(0.67-1.61) | 0.83(0.55-1.25) | 0.78(0.42-1.44) |
| **Lymphoma/Leukemia** |  |  |  |  |
| T1 | 1.31(0.52-3.32) | 1.20(0.49-2.89) | 1.72(0.71-4.19) | 1.39(0.43-4.44) |
| T2(ref group) | 1.00(reference) | 1.00(reference) | 1.00(reference) | 1.00(reference) |
| T3 | 0.96(0.41-2.25) | 1.40(0.52-3.75) | 1.31(0.52-3.27) | 1.34(0.48-3.77) |
| HR for trend | 0.86(0.53-1.39) | 1.06(0.66-1.70) | 0.86(0.56-1.34) | 1.02(0.52-2.00) |
| **Urinary system** |  |  |  |  |
| T1 | 1.50(0.78-2.91) | 0.68(0.36-1.28) | 1.01(0.52-1.93) | 1.19(0.55-2.55) |
| T2(ref group) | 1.00(reference) | 1.00(reference) | 1.00(reference) | 1.00(reference) |
| T3 | 0.87(0.45-1.68) | 1.00(0.52-1.92) | 1.06(0.57-1.98) | 0.94(0.45-1.96) |
| HR for trend | 0.76(0.53-1.09) | 1.21(0.87-1.71) | 1.03(0.74-1.43) | 0.89(0.55-1.44) |
| **Skin** |  |  |  |  |
| T1 | 0.90(0.40-2.05) | 0.65(0.33-1.26) | 0.54(0.24-1.22) | 0.68(0.29-1.58) |
| T2(ref group) | 1.00(reference) | 1.00(reference) | 1.00(reference) | 1.00(reference) |
| T3 | 2.21(1.12-4.38)* | 1.01(0.52-1.96) | 1.58(0.85-2.92) | 1.87(0.89-3.89) |
| HR for trend | 1.65(1.11-2.47)* | 1.25(0.88-1.77) | 1.69(1.17-2.43)* | 1.68(1.03-2.73)* |
| **Cervix/Uterus** |  |  |  |  |
| T1 | 0.94(0.53-1.67) | 0.81(0.47-1.39) | 0.90(0.50-1.60) | 1.04(0.57-1.88) |
| T2(ref group) | 1.00(reference) | 1.00(reference) | 1.00(reference) | 1.00(reference) |
| T3 | 1.09(0.63-1.90) | 0.96(0.56-1.66) | 1.29(0.76-2.19) | 0.96(0.51-1.81) |
| HR for trend | 1.08(0.79-1.47) | 1.09(0.82-1.44) | 1.21(0.91-1.60) | 0.96(0.65-1.42) |
| **Other sites** |  |  |  |  |
| T1 | 1.01(0.74-1.38) | 0.84(0.65-1.10) | 1.07(0.80-1.43) | 0.85(0.61-1.17) |
| T2(ref group) | 1.00(reference) | 1.00(reference) | 1.00(reference) | 1.00(reference) |
| T3 | 1.30(0.99-1.72) | 0.77(0.57-1.04) | 1.17(0.89-1.55) | 1.06(0.78-1.45) |
| HR for trend | 1.15(0.98-1.35) | 0.97(0.84-1.13) | 1.05(0.91-1.21) | 1.12(0.91-1.37) |

Abbreviation: BMI, body mass index; BSA, body surface area; BFP, body fat percentage.

Continuous body measurements were divided into tertiles based on gender-specific percentages, denoted as T1, T2, and T3 from low to high. T2 was set as the reference group.

* P<0.05.

Table S7. Difference in Hazard ratios among body measurements in cancer risk for female.

| **Cancer Sites** | **HEIGHT vs. BMI** | **BSA vs. BMI** | **BFP vs. BMI** | **BSA vs. HEIGHT** | **BFP vs. HEIGHT** | **BSA vs. BFP** |
| --- | --- | --- | --- | --- | --- | --- |
| **Overall cancer** |  |  |  |  |  |  |
| Low level (T1) | 0.14 | 0.47 | 0.14 | 0.12 | 0.45 | 0.12 |
| High level (T3) | 0.2 | 0.33 | 0.26 | 0.1 | 0.43 | 0.14 |
| Per level increased | 0.39 | 0.36 | 0.35 | 0.47 | 0.43 | 0.46 |
| **Lung** |  |  |  |  |  |  |
| Low level (T1) | 0.42 | 0.28 | 0.25 | 0.2 | 0.29 | 0.11 |
| High level (T3) | 0.47 | 0.09 | 0.47 | 0.09 | 0.5 | 0.09 |
| Per level increased | 0.36 | 0.22 | 0.33 | 0.34 | 0.44 | 0.43 |
| **Breast** |  |  |  |  |  |  |
| Low level (T1) | 0.09 | 0.32 | 0.25 | 0.19 | 0.27 | 0.41 |
| High level (T3) | 0.08 | 0.27 | 0.21 | 0.22 | 0.31 | 0.41 |
| Per level increased | 0.48 | 0.44 | 0.48 | 0.42 | 0.47 | 0.46 |
| **Thyroid** |  |  |  |  |  |  |
| Low level (T1) | 0.07 | 0.2 | 0.32 | 0.27 | <0.05 | 0.09 |
| High level (T3) | <0.05 | 0.1 | 0.43 | 0.21 | <0.05 | 0.16 |
| Per level increased | 0.29 | 0.33 | 0.28 | 0.46 | 0.46 | 0.42 |
| **Stomach** |  |  |  |  |  |  |
| Low level (T1) | 0.47 | 0.13 | 0.46 | 0.12 | 0.48 | 0.13 |
| High level (T3) | 0.1 | 0.25 | 0.33 | <0.05 | 0.22 | 0.14 |
| Per level increased | 0.13 | 0.34 | 0.39 | 0.22 | 0.25 | 0.48 |
| **Colorectal** |  |  |  |  |  |  |
| Low level (T1) | 0.15 | 0.43 | 0.44 | 0.19 | 0.14 | 0.38 |
| High level (T3) | 0.28 | 0.45 | 0.37 | 0.32 | 0.4 | 0.41 |
| Per level increased | 0.38 | 0.48 | 0.36 | 0.36 | 0.26 | 0.36 |
| **Liver** |  |  |  |  |  |  |
| Low level (T1) | 0.09 | 0.41 | 0.31 | <0.05 | <0.05 | 0.38 |
| High level (T3) | 0.13 | 0.43 | 0.43 | 0.17 | 0.2 | 0.49 |
| Per level increased | 0.44 | 0.29 | 0.27 | 0.23 | 0.23 | 0.43 |
| **Lymphoma/Leukemia** |  |  |  |  |  |  |
| Low level (T1) | 0.44 | 0.34 | 0.47 | 0.28 | 0.42 | 0.39 |
| High level (T3) | 0.29 | 0.31 | 0.31 | 0.46 | 0.48 | 0.48 |
| Per level increased | 0.28 | 0.5 | 0.34 | 0.27 | 0.47 | 0.34 |
| **Urinary system** |  |  |  |  |  |  |
| Low level (T1) | <0.05 | 0.2 | 0.32 | 0.2 | 0.14 | 0.37 |
| High level (T3) | 0.38 | 0.33 | 0.44 | 0.45 | 0.45 | 0.4 |
| Per level increased | <0.05 | 0.11 | 0.3 | 0.24 | 0.15 | 0.32 |
| **Skin** |  |  |  |  |  |  |
| Low level (T1) | 0.27 | 0.19 | 0.32 | 0.37 | 0.46 | 0.35 |
| High level (T3) | 0.05 | 0.23 | 0.37 | 0.17 | 0.11 | 0.37 |
| Per level increased | 0.15 | 0.47 | 0.48 | 0.12 | 0.16 | 0.5 |
| **Cervix/Uterus** |  |  |  |  |  |  |
| Low level (T1) | 0.36 | 0.46 | 0.41 | 0.4 | 0.27 | 0.37 |
| High level (T3) | 0.37 | 0.33 | 0.38 | 0.22 | 0.5 | 0.24 |
| Per level increased | 0.48 | 0.3 | 0.32 | 0.31 | 0.31 | 0.18 |
| **Other sites** |  |  |  |  |  |  |
| Low level (T1) | 0.19 | 0.39 | 0.22 | 0.12 | 0.5 | 0.14 |
| High level (T3) | <0.05 | 0.3 | 0.17 | <0.05 | 0.07 | 0.31 |
| Per level increased | 0.07 | 0.21 | 0.42 | 0.23 | 0.14 | 0.31 |

The difference of hazard ratios (HRs) in each group were tested using two-sample Z-test.

Table S8. Relationship between body measurements and cancer risk in male and female group by median.

| **Cancer Sites** | **BMI** | **Height** | **BSA** | **BFP** |
| --- | --- | --- | --- | --- |
| **Male** |  |  |  |  |
| **Overall cancer** |  |  |  |  |
| ≤Median | 1.00(reference) | 1.00(reference) | 1.00(reference) | 1.00(reference) |
| >Median | 1.08(0.97-1.20) | 1.21(1.09-1.34)* | 1.16(1.05-1.29)* | 1.10(0.97-1.25) |
| **Lung** |  |  |  |  |
| ≤Median | 1.00(reference) | 1.00(reference) | 1.00(reference) | 1.00(reference) |
| >Median | 0.88(0.69-1.11) | 1.01(0.80-1.28) | 0.99(0.78-1.26) | 0.89(0.67-1.19) |
| **Breast** |  |  |  |  |
| ≤Median | 1.00(reference) | 1.00(reference) | 1.00(reference) | 1.00(reference) |
| >Median | 1.34(0.49-3.63) | 1.36(0.51-3.60) | 1.51(0.56-4.07) | 0.97(0.31-3.06) |
| **Thyroid** |  |  |  |  |
| ≤Median | 1.00(reference) | 1.00(reference) | 1.00(reference) | 1.00(reference) |
| >Median | 1.68(1.12-2.52)* | 1.53(1.04-2.23)* | 1.64(1.10-2.44)* | 1.36(0.88-2.10) |
| **Stomach** |  |  |  |  |
| ≤Median | 1.00(reference) | 1.00(reference) | 1.00(reference) | 1.00(reference) |
| >Median | 1.29(0.93-1.79) | 1.26(0.91-1.74) | 1.40(1.01-1.95)* | 1.63(1.06-2.49)* |
| **Colorectal** |  |  |  |  |
| ≤Median | 1.00(reference) | 1.00(reference) | 1.00(reference) | 1.00(reference) |
| >Median | 1.06(0.76-1.48) | 1.21(0.86-1.69) | 1.12(0.80-1.57) | 1.43(0.93-2.20) |
| **Liver** |  |  |  |  |
| ≤Median | 1.00(reference) | 1.00(reference) | 1.00(reference) | 1.00(reference) |
| >Median | 1.07(0.76-1.51) | 1.05(0.74-1.48) | 0.83(0.59-1.19) | 0.97(0.64-1.47) |
| **Lymphoma/Leukemia** |  |  |  |  |
| ≤Median | 1.00(reference) | 1.00(reference) | 1.00(reference) | 1.00(reference) |
| >Median | 1.15(0.77-1.72) | 1.24(0.83-1.85) | 1.25(0.84-1.87) | 1.10(0.67-1.83) |
| **Urinary system** |  |  |  |  |
| ≤Median | 1.00(reference) | 1.00(reference) | 1.00(reference) | 1.00(reference) |
| >Median | 1.20(0.75-1.91) | 1.68(1.06-2.65)* | 1.51(0.95-2.41) | 1.10(0.63-1.91) |
| **Skin** |  |  |  |  |
| ≤Median | 1.00(reference) | 1.00(reference) | 1.00(reference) | 1.00(reference) |
| >Median | 1.55(0.88-2.71) | 1.84(1.06-3.20)* | 1.99(1.13-3.50)* | 1.54(0.82-2.88) |
| **Prostate** |  |  |  |  |
| ≤Median | 1.00(reference) | 1.00(reference) | 1.00(reference) | 1.00(reference) |
| >Median | 0.96(0.56-1.66) | 1.39(0.79-2.43) | 0.74(0.41-1.33) | 0.63(0.31-1.31) |
| **Other sites** |  |  |  |  |
| ≤Median | 1.00(reference) | 1.00(reference) | 1.00(reference) | 1.00(reference) |
| >Median | 1.01(0.81-1.26) | 1.17(0.94-1.45) | 1.20(0.96-1.49) | 1.09(0.83-1.41) |
| **Female** |  |  |  |  |
| **Overall cancer** |  |  |  |  |
| ≤Median | 1.00(reference) | 1.00(reference) | 1.00(reference) | 1.00(reference) |
| >Median | 1.14(1.01-1.27)* | 1.13(1.02-1.26)* | 1.17(1.05-1.30)* | 1.20(1.05-1.38)* |
| **Lung** |  |  |  |  |
| ≤Median | 1.00(reference) | 1.00(reference) | 1.00(reference) | 1.00(reference) |
| >Median | 0.91(0.66-1.24) | 1.19(0.88-1.60) | 1.14(0.86-1.52) | 1.20(0.81-1.80) |
| **Breast** |  |  |  |  |
| ≤Median | 1.00(reference) | 1.00(reference) | 1.00(reference) | 1.00(reference) |
| >Median | 1.10(0.88-1.39) | 1.09(0.88-1.34) | 1.23(0.99-1.51) | 1.14(0.87-1.49) |
| **Thyroid** |  |  |  |  |
| ≤Median | 1.00(reference) | 1.00(reference) | 1.00(reference) | 1.00(reference) |
| >Median | 1.45(1.09-1.93)* | 1.44(1.11-1.88)* | 1.34(1.03-1.74)* | 1.42(1.02-1.97)* |
| **Stomach** |  |  |  |  |
| ≤Median | 1.00(reference) | 1.00(reference) | 1.00(reference) | 1.00(reference) |
| >Median | 1.07(0.62-1.83) | 1.06(0.63-1.77) | 0.82(0.50-1.34) | 0.92(0.47-1.81) |
| **Colorectal** |  |  |  |  |
| ≤Median | 1.00(reference) | 1.00(reference) | 1.00(reference) | 1.00(reference) |
| >Median | 1.22(0.70-2.13) | 1.07(0.63-1.83) | 0.77(0.46-1.27) | 1.43(0.71-2.88) |
| **Liver** |  |  |  |  |
| ≤Median | 1.00(reference) | 1.00(reference) | 1.00(reference) | 1.00(reference) |
| >Median | 0.97(0.47-2.03) | 0.83(0.41-1.71) | 0.66(0.34-1.28) | 0.46(0.18-1.17) |
| **Lymphoma/Leukemia** |  |  |  |  |
| ≤Median | 1.00(reference) | 1.00(reference) | 1.00(reference) | 1.00(reference) |
| >Median | 0.91(0.42-1.96) | 0.99(0.46-2.12) | 0.93(0.46-1.89) | 0.58(0.21-1.61) |
| **Urinary system** |  |  |  |  |
| ≤Median | 1.00(reference) | 1.00(reference) | 1.00(reference) | 1.00(reference) |
| >Median | 0.80(0.45-1.42) | 1.31(0.75-2.26) | 1.06(0.62-1.80) | 0.85(0.41-1.75) |
| **Skin** |  |  |  |  |
| ≤Median | 1.00(reference) | 1.00(reference) | 1.00(reference) | 1.00(reference) |
| >Median | 1.91(1.01-3.61)* | 1.28(0.73-2.27) | 2.74(1.46-5.11)* | 2.09(1.00-4.37) |
| **Cervix/Uterus** |  |  |  |  |
| ≤Median | 1.00(reference) | 1.00(reference) | 1.00(reference) | 1.00(reference) |
| >Median | 0.90(0.55-1.47) | 1.26(0.79-1.99) | 1.55(0.97-2.47) | 0.86(0.48-1.56) |
| **Other sites** |  |  |  |  |
| ≤Median | 1.00(reference) | 1.00(reference) | 1.00(reference) | 1.00(reference) |
| >Median | 1.30(1.01-1.69)* | 0.97(0.76-1.23) | 1.16(0.92-1.47) | 1.55(1.14-2.10)* |

Abbreviation: BMI, body mass index; BSA, body surface area; BFP, body fat percentage.

Continuous body measurements were transformed into binary variables by median. ≤Median was set as the reference group.

* P<0.05.

Table S9. Difference in Hazard ratios among body measurements in CVD risk.

| **CVD** | **HEIGHT vs. BMI** | **BSA vs. BMI** | **BFP vs. BMI** | **BSA vs. HEIGHT** | **BFP vs. HEIGHT** | **BSA vs. BFP** |
| --- | --- | --- | --- | --- | --- | --- |
| **Overall CVD** |  |  |  |  |  |  |
| Low level (T1) | 0.07 | 0.1 | 0.07 | 0.44 | <0.05 | <0.05 |
| High level (T3) | 0.29 | 0.48 | <0.05 | 0.31 | <0.05 | <0.05 |
| Per level increased | <0.05 | 0.11 | <0.05 | 0.26 | <0.05 | <0.05 |
| **Myocardial infarction** |  |  |  |  |  |  |
| Low level (T1) | <0.05 | <0.05 | 0.11 | 0.43 | <0.05 | <0.05 |
| High level (T3) | 0.23 | 0.22 | <0.05 | 0.5 | <0.05 | <0.05 |
| Per level increased | <0.05 | 0.06 | <0.05 | 0.36 | <0.05 | <0.05 |
| **Heart failure** |  |  |  |  |  |  |
| Low level (T1) | 0.2 | 0.17 | 0.2 | 0.45 | 0.4 | 0.44 |
| High level (T3) | 0.14 | 0.38 | 0.31 | 0.09 | 0.07 | 0.42 |
| Per level increased | 0.4 | 0.13 | 0.1 | 0.09 | 0.07 | 0.35 |
| **Stroke** |  |  |  |  |  |  |
| Low level (T1) | 0.2 | 0.11 | 0.18 | 0.34 | 0.06 | <0.05 |
| High level (T3) | 0.42 | 0.46 | 0.25 | 0.46 | 0.22 | 0.23 |
| Per level increased | 0.18 | 0.11 | 0.11 | 0.38 | <0.05 | <0.05 |

The difference of hazard ratios (HRs) in each group were tested using two-sample Z-test.

Table S10. Relationship between body measurements and risk of CVD group by median.

| **CVD** | **BMI** | **Height** | **BSA** | **BFP** |
| --- | --- | --- | --- | --- |
| **Overall CVD** |  |  |  |  |
| ≤Median | 1.00(reference) | 1.00(reference) | 1.00(reference) | 1.00(reference) |
| >Median | 1.21(1.13-1.30)* | 1.06(0.98-1.13) | 1.13(1.06-1.21)* | 1.35(1.23-1.49)* |
| **Myocardial infarction** |  |  |  |  |
| ≤Median | 1.00(reference) | 1.00(reference) | 1.00(reference) | 1.00(reference) |
| >Median | 1.40(1.24-1.59)* | 1.15(1.02-1.30)* | 1.18(1.06-1.33)* | 1.64(1.39-1.93)* |
| **Heart failure** |  |  |  |  |
| ≤Median | 1.00(reference) | 1.00(reference) | 1.00(reference) | 1.00(reference) |
| >Median | 1.29(1.08-1.53)* | 1.22(1.02-1.45)* | 1.47(1.24-1.74)* | 1.48(1.15-1.91)* |
| **Stroke** |  |  |  |  |
| ≤Median | 1.00(reference) | 1.00(reference) | 1.00(reference) | 1.00(reference) |
| >Median | 1.09(0.99-1.19) | 0.96(0.87-1.05) | 1.02(0.93-1.11) | 1.24(1.09-1.40)* |

Table S11. Relationship between body measurements and risk of CVD in male and female.

| **CVD** | **BMI** | **Height** | **BSA** | **BFP** |
| --- | --- | --- | --- | --- |
| **Male** |  |  |  |  |
| **Overall CVD** |  |  |  |  |
| T1 | 0.90(0.81-1.00) | 0.97(0.89-1.07) | 0.96(0.87-1.05) | 0.82(0.71-0.95)* |
| T2(ref group) | 1.00(reference) | 1.00(reference) | 1.00(reference) | 1.00(reference) |
| T3 | 1.11(1.01-1.21)* | 1.03(0.92-1.15) | 1.09(0.99-1.21) | 1.20(1.08-1.33)* |
| HR for trend | 1.11(1.05-1.17)* | 1.03(0.98-1.08) | 1.07(1.02-1.13)* | 1.20(1.12-1.29)* |
| **Myocardial infarction** |  |  |  |  |
| T1 | 0.78(0.65-0.94)* | 1.13(0.96-1.33) | 0.96(0.81-1.13) | 0.62(0.48-0.80)* |
| T2(ref group) | 1.00(reference) | 1.00(reference) | 1.00(reference) | 1.00(reference) |
| T3 | 1.19(1.03-1.39)* | 1.24(1.04-1.49)* | 1.18(1.00-1.39)* | 1.46(1.23-1.74)* |
| HR for trend | 1.23(1.13-1.35)* | 1.03(0.95-1.13) | 1.11(1.02-1.21)* | 1.51(1.34-1.71)* |
| **Heart failure** |  |  |  |  |
| T1 | 0.96(0.75-1.24) | 0.80(0.64-1.01) | 0.79(0.62-1.01) | 0.82(0.56-1.21) |
| T2(ref group) | 1.00(reference) | 1.00(reference) | 1.00(reference) | 1.00(reference) |
| T3 | 1.17(0.92-1.48) | 1.06(0.81-1.39) | 1.24(0.97-1.58) | 1.26(0.97-1.63) |
| HR for trend | 1.11(0.97-1.26) | 1.16(1.02-1.32)* | 1.25(1.10-1.42)* | 1.24(1.04-1.49)* |
| **Stroke** |  |  |  |  |
| T1 | 0.95(0.83-1.08) | 0.97(0.86-1.10) | 1.05(0.92-1.18) | 0.92(0.77-1.11) |
| T2(ref group) | 1.00(reference) | 1.00(reference) | 1.00(reference) | 1.00(reference) |
| T3 | 1.02(0.90-1.15) | 0.94(0.81-1.09) | 1.03(0.90-1.18) | 1.05(0.92-1.20) |
| HR for trend | 1.03(0.97-1.11) | 0.99(0.92-1.06) | 0.99(0.92-1.06) | 1.06(0.97-1.17) |
| **Female** |  |  |  |  |
| **Overall CVD** |  |  |  |  |
| T1 | 0.79(0.66-0.95)* | 0.99(0.86-1.13) | 0.94(0.81-1.09) | 0.67(0.52-0.87)* |
| T2(ref group) | 1.00(reference) | 1.00(reference) | 1.00(reference) | 1.00(reference) |
| T3 | 1.07(0.94-1.22) | 1.02(0.86-1.22) | 1.07(0.94-1.23) | 1.30(1.10-1.54)* |
| HR for trend | 1.15(1.05-1.25)* | 1.02(0.94-1.10) | 1.07(0.99-1.15) | 1.36(1.20-1.54)* |
| **Myocardial infarction** |  |  |  |  |
| T1 | 0.69(0.50-0.95)* | 0.83(0.65-1.07) | 1.09(0.83-1.42) | 0.72(0.48-1.10) |
| T2(ref group) | 1.00(reference) | 1.00(reference) | 1.00(reference) | 1.00(reference) |
| T3 | 0.90(0.72-1.14) | 0.96(0.71-1.28) | 1.18(0.92-1.52) | 1.44(1.07-1.94)* |
| HR for trend | 1.09(0.94-1.27) | 1.08(0.94-1.25) | 1.05(0.92-1.20) | 1.42(1.15-1.75)* |
| **Heart failure** |  |  |  |  |
| T1 | 0.71(0.44-1.16) | 0.85(0.61-1.19) | 0.79(0.54-1.17) | 0.59(0.29-1.23) |
| T2(ref group) | 1.00(reference) | 1.00(reference) | 1.00(reference) | 1.00(reference) |
| T3 | 1.44(1.04-2.01)* | 1.03(0.67-1.57) | 1.46(1.06-2.03)* | 1.67(1.08-2.58)* |
| HR for trend | 1.43(1.15-1.77)* | 1.11(0.91-1.35) | 1.37(1.15-1.64)* | 1.67(1.21-2.32)* |
| **Stroke** |  |  |  |  |
| T1 | 0.84(0.66-1.07) | 1.11(0.92-1.33) | 0.97(0.80-1.17) | 0.62(0.43-0.90)* |
| T2(ref group) | 1.00(reference) | 1.00(reference) | 1.00(reference) | 1.00(reference) |
| T3 | 1.05(0.89-1.25) | 1.08(0.85-1.38) | 0.99(0.83-1.18) | 1.18(0.94-1.47) |
| HR for trend | 1.10(0.99-1.23) | 0.97(0.87-1.09) | 1.01(0.92-1.11) | 1.30(1.10-1.54)* |

Abbreviation: BMI, body mass index; BSA, body surface area; BFP, body fat percentage.

Continuous body measurements were divided into tertiles based on gender-specific percentages, denoted as T1, T2, and T3 from low to high. T2 was set as the reference group.

* P<0.05.

Table S12. Difference in hazard ratios among body measurements in CVD risk for male and female.

| **CVD** | **HEIGHT vs. BMI** | **BSA vs. BMI** | **BFP vs. BMI** | **BSA vs. HEIGHT** | **BFP vs. HEIGHT** | **BSA vs. BFP** |
| --- | --- | --- | --- | --- | --- | --- |
| **Male** |  |  |  |  |  |  |
| **Overall CVD** |  |  |  |  |  |  |
| Low level (T1) | 0.15 | 0.21 | 0.16 | 0.41 | <0.05 | <0.05 |
| High level (T3) | 0.16 | 0.44 | 0.13 | 0.2 | <0.05 | 0.11 |
| Per level increased | <0.05 | 0.18 | <0.05 | 0.15 | <0.05 | <0.05 |
| **Myocardial infarction** |  |  |  |  |  |  |
| Low level (T1) | <0.05 | <0.05 | 0.08 | 0.09 | <0.05 | <0.05 |
| High level (T3) | 0.37 | 0.46 | 0.04 | 0.34 | 0.1 | <0.05 |
| Per level increased | <0.05 | <0.05 | <0.05 | 0.13 | <0.05 | <0.05 |
| **Heart failure** |  |  |  |  |  |  |
| Low level (T1) | 0.15 | 0.14 | 0.25 | 0.47 | 0.46 | 0.43 |
| High level (T3) | 0.3 | 0.38 | 0.34 | 0.21 | 0.19 | 0.46 |
| Per level increased | 0.3 | 0.09 | 0.15 | 0.21 | 0.27 | 0.48 |
| **Stroke** |  |  |  |  |  |  |
| Low level (T1) | 0.39 | 0.15 | 0.4 | 0.21 | 0.31 | 0.13 |
| High level (T3) | 0.2 | 0.44 | 0.35 | 0.18 | 0.12 | 0.41 |
| Per level increased | 0.17 | 0.19 | 0.3 | 0.47 | 0.09 | 0.1 |
| **Female** |  |  |  |  |  |  |
| **Overall CVD** |  |  |  |  |  |  |
| Low level (T1) | <0.05 | 0.07 | 0.17 | 0.32 | <0.05 | <0.05 |
| High level (T3) | 0.34 | 0.5 | 0.04 | 0.34 | <0.05 | <0.05 |
| Per level increased | <0.05 | 0.11 | <0.05 | 0.19 | <0.05 | <0.05 |
| **Myocardial infarction** |  |  |  |  |  |  |
| Low level (T1) | 0.18 | <0.05 | 0.43 | 0.08 | 0.28 | 0.05 |
| High level (T3) | 0.39 | 0.06 | <0.05 | 0.14 | <0.05 | 0.16 |
| Per level increased | 0.47 | 0.35 | <0.05 | 0.38 | <0.05 | <0.05 |
| **Heart failure** |  |  |  |  |  |  |
| Low level (T1) | 0.28 | 0.37 | 0.34 | 0.39 | 0.19 | 0.25 |
| High level (T3) | 0.11 | 0.48 | 0.3 | 0.1 | 0.06 | 0.32 |
| Per level increased | <0.05 | 0.39 | 0.21 | 0.06 | <0.05 | 0.15 |
| **Stroke** |  |  |  |  |  |  |
| Low level (T1) | <0.05 | 0.18 | 0.09 | 0.17 | <0.05 | <0.05 |
| High level (T3) | 0.42 | 0.31 | 0.22 | 0.27 | 0.31 | 0.12 |
| Per level increased | 0.06 | 0.11 | 0.06 | 0.32 | <0.05 | <0.05 |

The difference of hazard ratios (HRs) in each group were tested using two-sample Z-test.

Table S13. Relationship between body measurements and risk of CVD in male and female group by median.

| **CVD** | **BMI** | **Height** | **BSA** | **BFP** |
| --- | --- | --- | --- | --- |
| **Male** |  |  |  |  |
| **Overall CVD** |  |  |  |  |
| ≤Median | 1.00(reference) | 1.00(reference) | 1.00(reference) | 1.00(reference) |
| >Median | 1.26(1.16-1.37)* | 1.04(0.96-1.13) | 1.14(1.05-1.24)* | 1.30(1.17-1.46)* |
| **Myocardial infarction** |  |  |  |  |
| ≤Median | 1.00(reference) | 1.00(reference) | 1.00(reference) | 1.00(reference) |
| >Median | 1.52(1.32-1.76)* | 1.12(0.97-1.29) | 1.21(1.06-1.40)* | 1.70(1.40-2.06)* |
| **Heart failure** |  |  |  |  |
| ≤Median | 1.00(reference) | 1.00(reference) | 1.00(reference) | 1.00(reference) |
| >Median | 1.26(1.02-1.55)* | 1.15(0.93-1.43) | 1.41(1.15-1.73)* | 1.49(1.11-2.00)* |
| **Stroke** |  |  |  |  |
| ≤Median | 1.00(reference) | 1.00(reference) | 1.00(reference) | 1.00(reference) |
| >Median | 1.12(1.01-1.25)* | 0.95(0.85-1.06) | 1.03(0.93-1.15) | 1.14(0.99-1.31) |
| **Female** |  |  |  |  |
| **Overall CVD** |  |  |  |  |
| ≤Median | 1.00(reference) | 1.00(reference) | 1.00(reference) | 1.00(reference) |
| >Median | 1.24(1.08-1.42)* | 1.08(0.95-1.23) | 1.20(1.07-1.35)* | 1.64(1.35-2.01)* |
| **Myocardial infarction** |  |  |  |  |
| ≤Median | 1.00(reference) | 1.00(reference) | 1.00(reference) | 1.00(reference) |
| >Median | 1.29(1.01-1.64)* | 1.20(0.96-1.51) | 1.20(0.97-1.49) | 1.53(1.10-2.14)* |
| **Heart failure** |  |  |  |  |
| ≤Median | 1.00(reference) | 1.00(reference) | 1.00(reference) | 1.00(reference) |
| >Median | 1.50(1.07-2.12)* | 1.39(1.02-1.90)* | 1.71(1.27-2.30)* | 1.55(0.93-2.59) |
| **Stroke** |  |  |  |  |
| ≤Median | 1.00(reference) | 1.00(reference) | 1.00(reference) | 1.00(reference) |
| >Median | 1.16(0.97-1.38) | 0.97(0.82-1.16) | 1.09(0.93-1.27) | 1.90(1.43-2.52)* |

**Table 14. Interactions of body measurements stratified by sex for binary exposure in overall cancer and cardiovascular diseases.**

| **Disease** | **Sex** | **Subgroup**  **(vs. ≤Median)** | **BMI** | | **Height** | | **BSA** | | **BFP** | |
| --- | --- | --- | --- | --- | --- | --- | --- | --- | --- | --- |
|  |  |  | **HR(95%CI)** | ***P* value** | **HR(95%CI)** | ***P* value** | **HR(95%CI)** | ***P* value** | **HR(95%CI)** | ***P* value** |
| Cancer | Male | >Median | 1.08(0.97-1.20) | 0.26 | 1.21(1.09-1.34)* | 0.18 | 1.16(1.05-1.29)* | 0.47 | 1.10(0.97-1.25) | 0.19 |
|  | Female | >Median | 1.14(1.01-1.27)* |  | 1.13(1.02-1.26)* |  | 1.17(1.05-1.30)* |  | 1.20(1.05-1.38)* |  |
| CVD | Male | >Median | 1.26(1.16-1.37)* | 0.44 | 1.04(0.96-1.13) | 0.30 | 1.14(1.05-1.24)* | 0.26 | 1.30(1.17-1.46)* | 0.02 |
|  | Female | >Median | 1.24(1.08-1.42)* |  | 1.08(0.95-1.23) |  | 1.20(1.07-1.35)* |  | 1.64(1.35-2.01)* |  |

Abbreviation: CVD, Cardiovascular diseases; BMI, body mass index; BSA, body surface area; BFP, body fat percentage.

The difference of hazard ratios (HRs) in each group (interactions) were tested using two-sample Z-test.

*P<0.05

Table S15. Relationship between body measurements and cancer risk after excluded DM and CKD.

| **Cancer Sites** | **BMI** | **Height** | **BSA** | **BFP** |
| --- | --- | --- | --- | --- |
| **Overall cancer** |  |  |  |  |
| T1 | 0.98(0.89-1.08) | 0.86(0.79-0.94)* | 0.99(0.90-1.08) | 0.86(0.77-0.96)* |
| T2(ref group) | 1.00(reference) | 1.00(reference) | 1.00(reference) | 1.00(reference) |
| T3 | 1.07(0.97-1.17) | 1.08(0.98-1.19) | 1.18(1.08-1.30)* | 1.05(0.95-1.15) |
| HR for trend | 1.04(0.99-1.10) | 1.12(1.07-1.18)* | 1.10(1.04-1.15)* | 1.10(1.03-1.17)* |
| **Lung** |  |  |  |  |
| T1 | 1.16(0.92-1.45) | 0.84(0.68-1.04) | 1.01(0.81-1.26) | 0.89(0.68-1.18) |
| T2(ref group) | 1.00(reference) | 1.00(reference) | 1.00(reference) | 1.00(reference) |
| T3 | 1.03(0.82-1.29) | 0.96(0.75-1.22) | 1.11(0.88-1.40) | 0.83(0.66-1.05) |
| HR for trend | 0.94(0.83-1.06) | 1.08(0.96-1.21) | 1.05(0.93-1.18) | 0.95(0.81-1.10) |
| **Breast** |  |  |  |  |
| T1 | 0.82(0.63-1.07) | 0.63(0.49-0.80)* | 0.81(0.63-1.05) | 0.73(0.55-0.96)* |
| T2(ref group) | 1.00(reference) | 1.00(reference) | 1.00(reference) | 1.00(reference) |
| T3 | 1.00(0.78-1.28) | 0.74(0.58-0.95)* | 0.94(0.74-1.20) | 0.91(0.69-1.21) |
| HR for trend | 1.10(0.95-1.27) | 1.09(0.96-1.24) | 1.07(0.94-1.22) | 1.12(0.94-1.34) |
| **Thyroid** |  |  |  |  |
| T1 | 0.75(0.56-1.00) | 0.96(0.73-1.28) | 0.89(0.66-1.20) | 0.77(0.57-1.03) |
| T2(ref group) | 1.00(reference) | 1.00(reference) | 1.00(reference) | 1.00(reference) |
| T3 | 1.17(0.91-1.51) | 1.71(1.32-2.21)* | 1.62(1.26-2.08)* | 1.37(1.04-1.81)* |
| HR for trend | 1.24(1.07-1.44)* | 1.35(1.18-1.55)* | 1.37(1.20-1.58)* | 1.34(1.12-1.59)* |
| **Stomach** |  |  |  |  |
| T1 | 0.83(0.59-1.17) | 1.28(0.90-1.81) | 0.89(0.64-1.24) | 0.89(0.58-1.37) |
| T2(ref group) | 1.00(reference) | 1.00(reference) | 1.00(reference) | 1.00(reference) |
| T3 | 0.97(0.69-1.35) | 1.60(1.09-2.34)* | 1.07(0.75-1.53) | 1.24(0.87-1.76) |
| HR for trend | 1.08(0.90-1.29) | 1.09(0.92-1.31) | 1.10(0.92-1.31) | 1.19(0.94-1.50) |
| **Colorectal** |  |  |  |  |
| T1 | 0.74(0.52-1.05) | 0.90(0.64-1.25) | 0.82(0.59-1.16) | 0.53(0.33-0.85)* |
| T2(ref group) | 1.00(reference) | 1.00(reference) | 1.00(reference) | 1.00(reference) |
| T3 | 0.90(0.65-1.25) | 1.19(0.83-1.71) | 1.11(0.79-1.56) | 0.92(0.65-1.28) |
| HR for trend | 1.09(0.91-1.31) | 1.15(0.96-1.37) | 1.16(0.97-1.39) | 1.21(0.95-1.53) |
| **Liver** |  |  |  |  |
| T1 | 1.16(0.80-1.69) | 0.81(0.57-1.15) | 1.30(0.91-1.86) | 1.08(0.70-1.67) |
| T2(ref group) | 1.00(reference) | 1.00(reference) | 1.00(reference) | 1.00(reference) |
| T3 | 0.99(0.67-1.45) | 0.85(0.56-1.27) | 0.96(0.64-1.46) | 0.76(0.51-1.12) |
| HR for trend | 0.92(0.75-1.12) | 1.04(0.85-1.27) | 0.85(0.70-1.04) | 0.83(0.65-1.07) |
| **Lymphoma/Leukemia** |  |  |  |  |
| T1 | 0.88(0.56-1.38) | 0.69(0.46-1.05) | 0.76(0.49-1.17) | 1.00(0.56-1.79) |
| T2(ref group) | 1.00(reference) | 1.00(reference) | 1.00(reference) | 1.00(reference) |
| T3 | 1.00(0.65-1.53) | 0.89(0.56-1.41) | 0.95(0.62-1.47) | 1.51(0.94-2.42) |
| HR for trend | 1.06(0.84-1.35) | 1.15(0.92-1.45) | 1.12(0.89-1.41) | 1.27(0.94-1.73) |
| **Urinary system** |  |  |  |  |
| T1 | 0.98(0.62-1.54) | 0.88(0.57-1.37) | 0.97(0.62-1.52) | 0.91(0.54-1.52) |
| T2(ref group) | 1.00(reference) | 1.00(reference) | 1.00(reference) | 1.00(reference) |
| T3 | 1.05(0.67-1.63) | 1.19(0.76-1.88) | 1.18(0.76-1.84) | 1.09(0.68-1.74) |
| HR for trend | 1.04(0.81-1.32) | 1.16(0.92-1.47) | 1.10(0.87-1.39) | 1.09(0.81-1.48) |
| **Skin** |  |  |  |  |
| T1 | 0.96(0.56-1.64) | 0.84(0.51-1.38) | 0.75(0.43-1.29) | 0.69(0.39-1.20) |
| T2(ref group) | 1.00(reference) | 1.00(reference) | 1.00(reference) | 1.00(reference) |
| T3 | 1.96(1.22-3.15)* | 1.47(0.93-2.34) | 1.91(1.21-3.01)* | 1.79(1.09-2.93)* |
| HR for trend | 1.47(1.13-1.92)* | 1.33(1.04-1.71)* | 1.63(1.26-2.11)* | 1.63(1.19-2.23)* |
| **Cervix/Uterus** |  |  |  |  |
| T1 | 0.97(0.54-1.74) | 0.85(0.49-1.46) | 0.93(0.52-1.67) | 1.07(0.59-1.94) |
| T2(ref group) | 1.00(reference) | 1.00(reference) | 1.00(reference) | 1.00(reference) |
| T3 | 1.16(0.67-2.03) | 0.99(0.57-1.71) | 1.36(0.80-2.32) | 1.04(0.55-1.97) |
| HR for trend | 1.10(0.80-1.50) | 1.08(0.81-1.44) | 1.22(0.91-1.62) | 0.98(0.67-1.46) |
| **Prostate** |  |  |  |  |
| T1 | 1.87(0.89-3.94) | 1.22(0.60-2.49) | 1.18(0.59-2.36) | 0.67(0.21-2.08) |
| T2(ref group) | 1.00(reference) | 1.00(reference) | 1.00(reference) | 1.00(reference) |
| T3 | 1.54(0.71-3.33) | 1.66(0.71-3.86) | 1.31(0.59-2.89) | 0.66(0.33-1.33) |
| HR for trend | 0.89(0.61-1.29) | 1.11(0.76-1.63) | 1.03(0.71-1.51) | 0.87(0.53-1.45) |
| **Other sites** |  |  |  |  |
| T1 | 1.01(0.82-1.24) | 0.92(0.76-1.11) | 1.10(0.90-1.34) | 0.96(0.76-1.21) |
| T2(ref group) | 1.00(reference) | 1.00(reference) | 1.00(reference) | 1.00(reference) |
| T3 | 1.15(0.95-1.40) | 0.99(0.81-1.22) | 1.23(1.01-1.51)* | 1.21(0.98-1.49) |
| HR for trend | 1.07(0.96-1.19) | 1.04(0.94-1.15) | 1.06(0.96-1.17) | 1.13(0.99-1.29) |

Abbreviation: BMI, body mass index; BSA, body surface area; BFP, body fat percentage.

Table S16. Relationship between body measurements and cancer risk in male after excluding DM and CKD.

| **Cancer Sites** | **BMI** | **Height** | **BSA** | **BFP** |
| --- | --- | --- | --- | --- |
| **Overall cancer** |  |  |  |  |
| T1 | 0.97(0.85-1.11) | 0.92(0.81-1.05) | 1.02(0.90-1.17) | 0.88(0.75-1.03) |
| T2(ref group) | 1.00(reference) | 1.00(reference) | 1.00(reference) | 1.00(reference) |
| T3 | 1.08(0.95-1.23) | 1.20(1.05-1.37)* | 1.29(1.13-1.47)* | 1.09(0.95-1.24) |
| HR for trend | 1.05(0.98-1.13) | 1.14(1.06-1.22)* | 1.12(1.04-1.20)* | 1.11(1.02-1.21)* |
| **Lung** |  |  |  |  |
| T1 | 1.34(1.01-1.79)* | 0.89(0.68-1.18) | 1.07(0.81-1.42) | 1.02(0.72-1.44) |
| T2(ref group) | 1.00(reference) | 1.00(reference) | 1.00(reference) | 1.00(reference) |
| T3 | 1.10(0.81-1.49) | 1.07(0.79-1.46) | 1.04(0.76-1.43) | 0.80(0.60-1.08) |
| HR for trend | 0.90(0.77-1.05) | 1.10(0.94-1.28) | 0.98(0.84-1.15) | 0.88(0.73-1.06) |
| **Breast** |  |  |  |  |
| T1 | 0.31(0.08-1.23) | 0.21(0.06-0.80)* | 0.96(0.25-3.70) | 0.12(0.01-1.05) |
| T2(ref group) | 1.00(reference) | 1.00(reference) | 1.00(reference) | 1.00(reference) |
| T3 | 0.75(0.24-2.34) | 0.34(0.09-1.27) | 2.26(0.65-7.86) | 1.18(0.39-3.61) |
| HR for trend | 1.43(0.74-2.77) | 1.26(0.67-2.36) | 1.57(0.81-3.02) | 2.20(0.97-4.98) |
| **Thyroid** |  |  |  |  |
| T1 | 0.65(0.38-1.11) | 0.71(0.43-1.18) | 0.75(0.43-1.32) | 0.96(0.56-1.63) |
| T2(ref group) | 1.00(reference) | 1.00(reference) | 1.00(reference) | 1.00(reference) |
| T3 | 1.10(0.72-1.69) | 1.37(0.88-2.12) | 1.77(1.14-2.77)* | 1.62(1.01-2.60)* |
| HR for trend | 1.27(0.98-1.64) | 1.39(1.09-1.77)* | 1.58(1.21-2.05)* | 1.33(1.00-1.77) |
| **Stomach** |  |  |  |  |
| T1 | 0.73(0.49-1.10) | 1.39(0.91-2.12) | 1.02(0.68-1.53) | 0.73(0.42-1.26) |
| T2(ref group) | 1.00(reference) | 1.00(reference) | 1.00(reference) | 1.00(reference) |
| T3 | 1.01(0.67-1.51) | 1.59(0.99-2.55) | 1.38(0.89-2.15) | 1.24(0.82-1.87) |
| HR for trend | 1.17(0.94-1.46) | 1.03(0.83-1.28) | 1.15(0.92-1.43) | 1.29(0.98-1.70) |
| **Colorectal** |  |  |  |  |
| T1 | 0.75(0.50-1.14) | 0.87(0.58-1.29) | 0.90(0.60-1.34) | 0.50(0.28-0.89)* |
| T2(ref group) | 1.00(reference) | 1.00(reference) | 1.00(reference) | 1.00(reference) |
| T3 | 0.96(0.65-1.41) | 1.27(0.83-1.95) | 1.29(0.86-1.95) | 0.93(0.63-1.38) |
| HR for trend | 1.12(0.90-1.39) | 1.21(0.98-1.49) | 1.20(0.97-1.49) | 1.24(0.94-1.63) |
| **Liver** |  |  |  |  |
| T1 | 1.03(0.69-1.55) | 0.82(0.55-1.23) | 1.15(0.77-1.71) | 0.87(0.54-1.41) |
| T2(ref group) | 1.00(reference) | 1.00(reference) | 1.00(reference) | 1.00(reference) |
| T3 | 0.88(0.57-1.37) | 0.91(0.58-1.42) | 0.92(0.57-1.47) | 0.69(0.45-1.05) |
| HR for trend | 0.93(0.74-1.16) | 1.06(0.85-1.32) | 0.89(0.71-1.12) | 0.87(0.66-1.14) |
| **Lymphoma/Leukemia** | |  |  |  |
| T1 | 0.76(0.45-1.27) | 0.62(0.38-0.99)* | 0.59(0.36-0.97)* | 0.90(0.46-1.78) |
| T2(ref group) | 1.00(reference) | 1.00(reference) | 1.00(reference) | 1.00(reference) |
| T3 | 1.01(0.62-1.64) | 0.79(0.47-1.33) | 0.87(0.53-1.43) | 1.59(0.94-2.70) |
| HR for trend | 1.15(0.88-1.50) | 1.16(0.89-1.50) | 1.21(0.93-1.58) | 1.38(0.98-1.94) |
| **Urinary system** |  |  |  |  |
| T1 | 0.70(0.38-1.29) | 1.05(0.57-1.93) | 0.98(0.53-1.79) | 0.71(0.35-1.42) |
| T2(ref group) | 1.00(reference) | 1.00(reference) | 1.00(reference) | 1.00(reference) |
| T3 | 1.20(0.67-2.13) | 1.51(0.82-2.80) | 1.46(0.79-2.68) | 1.16(0.64-2.12) |
| *P* for trend | 0.10 | 0.24 | 0.22 | 0.22 |
| **Skin** |  |  |  |  |
| T1 | 1.04(0.52-2.08) | 1.02(0.50-2.11) | 1.08(0.53-2.23) | 0.73(0.35-1.55) |
| T2(ref group) | 1.00(reference) | 1.00(reference) | 1.00(reference) | 1.00(reference) |
| T3 | 1.59(0.83-3.05) | 1.95(1.01-3.75)* | 2.14(1.10-4.14)* | 1.67(0.87-3.24) |
| HR for trend | 1.26(0.88-1.78) | 1.41(1.01-1.98)* | 1.45(1.02-2.05)* | 1.53(1.02-2.30)* |
| **Prostate** |  |  |  |  |
| T1 | 1.87(0.89-3.94) | 1.22(0.60-2.49) | 1.18(0.59-2.36) | 0.67(0.21-2.08) |
| T2(ref group) | 1.00(reference) | 1.00(reference) | 1.00(reference) | 1.00(reference) |
| T3 | 1.54(0.71-3.33) | 1.66(0.71-3.86) | 1.31(0.59-2.89) | 0.66(0.33-1.33) |
| HR for trend | 0.89(0.61-1.29) | 1.11(0.76-1.63) | 1.03(0.71-1.51) | 0.87(0.53-1.45) |
| **Other sites** |  |  |  |  |
| T1 | 1.00(0.76-1.31) | 1.01(0.77-1.32) | 1.13(0.86-1.48) | 1.12(0.81-1.57) |
| T2(ref group) | 1.00(reference) | 1.00(reference) | 1.00(reference) | 1.00(reference) |
| T3 | 1.11(0.85-1.45) | 1.22(0.92-1.62) | 1.36(1.02-1.80)* | 1.40(1.05-1.86)* |
| HR for trend | 1.06(0.91-1.22) | 1.10(0.95-1.26) | 1.09(0.95-1.26) | 1.15(0.96-1.37) |

Abbreviation: BMI, body mass index; BSA, body surface area; BFP, body fat percentage.

Continuous body measurements were divided into tertiles based on gender-specific percentages, denoted as T1, T2, and T3 from low to high. T2 was set as the reference group.

Table S17. Relationship between body measurements and cancer risk in female after excluding DM and CKD.

| **Cancer Sites** | **BMI** | **Height** | **BSA** | **BFP** |
| --- | --- | --- | --- | --- |
| **Overall cancer** |  |  |  |  |
| T1 | 0.92(0.80-1.05) | 0.83(0.73-0.94)* | 0.90(0.79-1.03) | 0.83(0.72-0.96)* |
| T2(ref group) | 1.00(reference) | 1.00(reference) | 1.00(reference) | 1.00(reference) |
| T3 | 1.10(0.97-1.25) | 1.00(0.87-1.13) | 1.11(0.98-1.26) | 1.05(0.91-1.21) |
| HR for trend | 1.10(1.02-1.18)* | 1.10(1.03-1.17)* | 1.11(1.04-1.19)* | 1.12(1.02-1.23)* |
| **Lung** |  |  |  |  |
| T1 | 0.85(0.57-1.25) | 0.78(0.55-1.09) | 0.88(0.60-1.27) | 0.69(0.43-1.09) |
| T2(ref group) | 1.00(reference) | 1.00(reference) | 1.00(reference) | 1.00(reference) |
| T3 | 0.94(0.67-1.33) | 0.81(0.55-1.20) | 1.17(0.83-1.65) | 0.92(0.62-1.36) |
| HR for trend | 1.04(0.85-1.28) | 1.04(0.86-1.26) | 1.16(0.96-1.39) | 1.13(0.86-1.47) |
| **Breast** |  |  |  |  |
| T1 | 0.85(0.65-1.11) | 0.66(0.51-0.85)* | 0.80(0.62-1.04) | 0.76(0.58-1.01) |
| T2(ref group) | 1.00(reference) | 1.00(reference) | 1.00(reference) | 1.00(reference) |
| T3 | 1.02(0.79-1.32) | 0.77(0.60-1.00)* | 0.91(0.71-1.17) | 0.90(0.67-1.20) |
| HR for trend | 1.09(0.95-1.27) | 1.09(0.95-1.24) | 1.06(0.93-1.21) | 1.09(0.91-1.31) |
| **Thyroid** |  |  |  |  |
| T1 | 0.80(0.57-1.13) | 1.12(0.79-1.57) | 0.95(0.67-1.34) | 0.71(0.50-1.02) |
| T2(ref group) | 1.00(reference) | 1.00(reference) | 1.00(reference) | 1.00(reference) |
| T3 | 1.23(0.90-1.67) | 1.90(1.38-2.62)* | 1.56(1.15-2.12)* | 1.28(0.91-1.82) |
| HR for trend | 1.24(1.03-1.48)* | 1.33(1.13-1.57)* | 1.31(1.11-1.54)* | 1.34(1.08-1.67)* |
| **Stomach** |  |  |  |  |
| T1 | 1.03(0.55-1.93) | 1.08(0.57-2.03) | 0.63(0.35-1.14) | 1.19(0.58-2.46) |
| T2(ref group) | 1.00(reference) | 1.00(reference) | 1.00(reference) | 1.00(reference) |
| T3 | 0.95(0.52-1.74) | 1.63(0.86-3.08) | 0.68(0.38-1.23) | 1.28(0.63-2.60) |
| HR for trend | 0.96(0.68-1.35) | 1.23(0.89-1.69) | 1.04(0.76-1.42) | 1.04(0.66-1.63) |
| **Colorectal** |  |  |  |  |
| T1 | 0.69(0.35-1.34) | 1.02(0.56-1.86) | 0.67(0.36-1.26) | 0.61(0.28-1.36) |
| T2(ref group) | 1.00(reference) | 1.00(reference) | 1.00(reference) | 1.00(reference) |
| T3 | 0.83(0.47-1.50) | 1.03(0.52-2.04) | 0.83(0.46-1.50) | 0.90(0.46-1.75) |
| HR for trend | 1.07(0.76-1.52) | 1.00(0.72-1.40) | 1.10(0.80-1.51) | 1.16(0.73-1.85) |
| **Liver** |  |  |  |  |
| T1 | 1.85(0.71-4.83) | 0.72(0.33-1.57) | 2.10(0.88-4.98) | 2.76(0.90-8.46) |
| T2(ref group) | 1.00(reference) | 1.00(reference) | 1.00(reference) | 1.00(reference) |
| T3 | 1.39(0.58-3.32) | 0.62(0.23-1.65) | 1.18(0.47-2.96) | 1.28(0.44-3.71) |
| HR for trend | 0.90(0.56-1.44) | 0.98(0.62-1.55) | 0.74(0.48-1.13) | 0.71(0.37-1.34) |
| **Lymphoma/Leukemia** | |  |  |  |
| T1 | 1.47(0.57-3.82) | 1.03(0.42-2.53) | 1.83(0.71-4.69) | 1.43(0.44-4.62) |
| T2(ref group) | 1.00(reference) | 1.00(reference) | 1.00(reference) | 1.00(reference) |
| T3 | 0.96(0.39-2.34) | 1.39(0.52-3.74) | 1.50(0.58-3.89) | 1.16(0.40-3.31) |
| HR for trend | 0.81(0.50-1.34) | 1.15(0.71-1.86) | 0.90(0.57-1.42) | 0.92(0.46-1.84) |
| **Urinary system** |  |  |  |  |
| T1 | 1.49(0.76-2.94) | 0.72(0.38-1.36) | 0.95(0.49-1.84) | 1.25(0.57-2.72) |
| T2(ref group) | 1.00(reference) | 1.00(reference) | 1.00(reference) | 1.00(reference) |
| T3 | 0.86(0.43-1.70) | 0.89(0.45-1.78) | 0.91(0.47-1.75) | 0.94(0.44-2.03) |
| *P* for trend | 0.14 | 0.51 | 0.89 | 0.58 |
| **Skin** |  |  |  |  |
| T1 | 0.82(0.35-1.94) | 0.69(0.34-1.39) | 0.43(0.18-1.06) | 0.62(0.27-1.45) |
| T2(ref group) | 1.00(reference) | 1.00(reference) | 1.00(reference) | 1.00(reference) |
| T3 | 2.49(1.22-5.05)* | 1.08(0.55-2.13) | 1.68(0.89-3.17) | 1.96(0.93-4.16) |
| HR for trend | 1.84(1.21-2.79)* | 1.25(0.87-1.79) | 1.90(1.29-2.81)* | 1.80(1.10-2.94)* |
| **Cervix/Uterus** |  |  |  |  |
| T1 | 0.97(0.54-1.74) | 0.85(0.49-1.46) | 0.93(0.52-1.67) | 1.07(0.59-1.94) |
| T2(ref group) | 1.00(reference) | 1.00(reference) | 1.00(reference) | 1.00(reference) |
| T3 | 1.16(0.67-2.03) | 0.99(0.57-1.71) | 1.36(0.80-2.32) | 1.04(0.55-1.97) |
| HR for trend | 1.10(0.80-1.50) | 1.08(0.81-1.44) | 1.22(0.91-1.62) | 0.98(0.67-1.46) |
| **Other sites** |  |  |  |  |
| T1 | 0.97(0.71-1.33) | 0.86(0.65-1.13) | 1.03(0.77-1.38) | 0.83(0.59-1.15) |
| T2(ref group) | 1.00(reference) | 1.00(reference) | 1.00(reference) | 1.00(reference) |
| T3 | 1.25(0.94-1.66) | 0.80(0.59-1.08) | 1.13(0.85-1.50) | 1.06(0.77-1.46) |
| HR for trend | 1.14(0.97-1.35) | 0.98(0.84-1.14) | 1.05(0.91-1.22) | 1.13(0.92-1.39) |

Abbreviation: BMI, body mass index; BSA, body surface area; BFP, body fat percentage.

Continuous body measurements were divided into tertiles based on gender-specific percentages, denoted as T1, T2, and T3 from low to high. T2 was set as the reference group.

Table S18. Relationship between body measurements and risk of CVD after excluding DM and CKD.

| **CVD** | **BMI** | **Height** | **BSA** | **BFP** |
| --- | --- | --- | --- | --- |
| **Overall CVD** |  |  |  |  |
| T1 | 0.89(0.81-0.97)* | 0.96(0.89-1.05) | 0.96(0.88-1.04) | 0.80(0.70-0.91)* |
| T2(ref group) | 1.00(reference) | 1.00(reference) | 1.00(reference) | 1.00(reference) |
| T3 | 1.07(0.98-1.16) | 1.04(0.94-1.15) | 1.07(0.99-1.17) | 1.22(1.11-1.33)* |
| HR for trend | 1.09(1.05-1.15)* | 1.04(0.99-1.09) | 1.06(1.01-1.11)* | 1.23(1.15-1.31)* |
| **Myocardial infarction** | |  |  |  |
| T1 | 0.76(0.64-0.89)* | 1.00(0.87-1.16) | 0.97(0.84-1.13) | 0.68(0.54-0.85)* |
| T2(ref group) | 1.00(reference) | 1.00(reference) | 1.00(reference) | 1.00(reference) |
| T3 | 1.06(0.93-1.21) | 1.14(0.97-1.34) | 1.14(0.98-1.31) | 1.49(1.27-1.74)* |
| HR for trend | 1.17(1.08-1.26)* | 1.06(0.98-1.14) | 1.08(1.00-1.17)* | 1.48(1.33-1.65)* |
| **Heart failure** |  |  |  |  |
| T1 | 0.95(0.75-1.21) | 0.82(0.67-1.01) | 0.78(0.63-0.98)* | 0.73(0.51-1.05) |
| T2(ref group) | 1.00(reference) | 1.00(reference) | 1.00(reference) | 1.00(reference) |
| T3 | 1.32(1.07-1.63)* | 1.01(0.79-1.30) | 1.29(1.04-1.59)* | 1.33(1.05-1.69)* |
| HR for trend | 1.19(1.06-1.34)* | 1.12(1.00-1.26) | 1.28(1.14-1.43)* | 1.34(1.14-1.59)* |
| **Stroke** |  |  |  |  |
| T1 | 0.95(0.84-1.07) | 0.99(0.88-1.10) | 1.06(0.95-1.19) | 0.88(0.74-1.05) |
| T2(ref group) | 1.00(reference) | 1.00(reference) | 1.00(reference) | 1.00(reference) |
| T3 | 1.00(0.89-1.11) | 1.01(0.88-1.15) | 1.03(0.92-1.16) | 1.06(0.94-1.19) |
| HR for trend | 1.02(0.96-1.08) | 1.01(0.95-1.08) | 0.98(0.93-1.04) | 1.08(1.00-1.18) |

Abbreviation: BMI, body mass index; BSA, body surface area; BFP, body fat percentage.

Continuous body measurements were divided into tertiles based on gender-specific percentages, denoted as T1, T2, and T3 from low to high. T2 was set as the reference group.

Table S19. Relationship between body measurements and risk of CVD in male and female after excluding DM and CKD.

| **CVD** | **BMI** | **Height** | **BSA** | **BFP** |
| --- | --- | --- | --- | --- |
| **Male** |  |  |  |  |
| **Overall CVD** |  |  |  |  |
| T1 | 0.89(0.80-1.00)* | 0.97(0.88-1.08) | 0.94(0.85-1.04) | 0.83(0.71-0.96)* |
| T2(ref group) | 1.00(reference) | 1.00(reference) | 1.00(reference) | 1.00(reference) |
| T3 | 1.09(0.99-1.21) | 1.04(0.93-1.17) | 1.08(0.97-1.21) | 1.20(1.08-1.34)* |
| HR for trend | 1.10(1.04-1.17)* | 1.03(0.98-1.09) | 1.07(1.01-1.13)* | 1.20(1.12-1.30)* |
| **Myocardial infarction** |  |  |  |  |
| T1 | 0.76(0.63-0.92)* | 1.14(0.96-1.36) | 0.90(0.75-1.07) | 0.65(0.50-0.86)* |
| T2(ref group) | 1.00(reference) | 1.00(reference) | 1.00(reference) | 1.00(reference) |
| T3 | 1.16(0.99-1.37) | 1.25(1.03-1.52)* | 1.12(0.94-1.34) | 1.49(1.24-1.79)* |
| HR for trend | 1.23(1.12-1.35)* | 1.03(0.94-1.13) | 1.12(1.02-1.23)* | 1.50(1.32-1.71)* |
| **Heart failure** |  |  |  |  |
| T1 | 1.00(0.75-1.32) | 0.76(0.59-0.98)* | 0.76(0.58-0.99)* | 0.78(0.52-1.18) |
| T2(ref group) | 1.00(reference) | 1.00(reference) | 1.00(reference) | 1.00(reference) |
| T3 | 1.24(0.95-1.62) | 0.95(0.71-1.29) | 1.18(0.89-1.55) | 1.25(0.94-1.66) |
| HR for trend | 1.12(0.97-1.29) | 1.14(0.99-1.31) | 1.25(1.08-1.44)* | 1.26(1.04-1.53)* |
| **Stroke** |  |  |  |  |
| T1 | 0.96(0.83-1.10) | 0.97(0.85-1.10) | 1.08(0.95-1.24) | 0.93(0.76-1.13) |
| T2(ref group) | 1.00(reference) | 1.00(reference) | 1.00(reference) | 1.00(reference) |
| T3 | 1.00(0.87-1.14) | 0.97(0.83-1.14) | 1.07(0.92-1.23) | 1.04(0.91-1.20) |
| HR for trend | 1.02(0.95-1.10) | 1.01(0.94-1.08) | 0.99(0.92-1.06) | 1.06(0.96-1.16) |
| **Female** |  |  |  |  |
| **Overall CVD** |  |  |  |  |
| T1 | 0.79(0.66-0.96)* | 0.95(0.82-1.10) | 0.96(0.82-1.12) | 0.69(0.53-0.90)* |
| T2(ref group) | 1.00(reference) | 1.00(reference) | 1.00(reference) | 1.00(reference) |
| T3 | 1.08(0.94-1.25) | 1.02(0.85-1.22) | 1.11(0.96-1.28) | 1.34(1.13-1.60)* |
| HR for trend | 1.15(1.05-1.26)* | 1.04(0.95-1.13) | 1.08(1.00-1.16) | 1.38(1.21-1.57)* |
| **Myocardial infarction** |  |  |  |  |
| T1 | 0.70(0.50-0.97)* | 0.78(0.60-1.00) | 1.14(0.86-1.51) | 0.74(0.49-1.12) |
| T2(ref group) | 1.00(reference) | 1.00(reference) | 1.00(reference) | 1.00(reference) |
| T3 | 0.92(0.72-1.18) | 0.92(0.68-1.25) | 1.24(0.95-1.61) | 1.55(1.14-2.11)* |
| HR for trend | 1.10(0.94-1.28) | 1.11(0.96-1.28) | 1.05(0.92-1.21) | 1.47(1.19-1.83)* |
| **Heart failure** |  |  |  |  |
| T1 | 0.73(0.44-1.21) | 0.94(0.65-1.36) | 0.81(0.53-1.22) | 0.55(0.26-1.14) |
| T2(ref group) | 1.00(reference) | 1.00(reference) | 1.00(reference) | 1.00(reference) |
| T3 | 1.50(1.05-2.13)* | 1.15(0.74-1.80) | 1.52(1.07-2.16)* | 1.63(1.05-2.55)* |
| HR for trend | 1.45(1.16-1.82)* | 1.10(0.89-1.36) | 1.39(1.15-1.68)* | 1.69(1.21-2.37)* |
| **Stroke** |  |  |  |  |
| T1 | 0.84(0.65-1.08) | 1.04(0.86-1.27) | 0.97(0.79-1.18) | 0.66(0.45-0.96)* |
| T2(ref group) | 1.00(reference) | 1.00(reference) | 1.00(reference) | 1.00(reference) |
| T3 | 1.06(0.88-1.27) | 1.07(0.84-1.38) | 1.02(0.84-1.23) | 1.19(0.95-1.51) |
| HR for trend | 1.11(0.99-1.24) | 1.01(0.90-1.13) | 1.02(0.93-1.13) | 1.29(1.08-1.54)* |

Abbreviation: BMI, body mass index; BSA, body surface area; BFP, body fat percentage.

Continuous body measurements were divided into tertiles based on gender-specific percentages, denoted as T1, T2, and T3 from low to high. T2 was set as the reference group.

Table20. Predictive performance of body measurements on overall cancer and CVD.

| **Outcome** | **Model** | **BMI** | | **Height** | | **BSA** | | **BFP** | |
| --- | --- | --- | --- | --- | --- | --- | --- | --- | --- |
|  |  | **AUC** | **95%CI** | **AUC** | **95%CI** | **AUC** | **95%CI** | **AUC** | **95%CI** |
| Cancer | Univariate | 0.517 | 0.507-0.528 | 0.515 | 0.505-0.526 | 0.506 | 0.496-0.517 | 0.587 | 0.577-0.597 |
| Cancer | Multivariate | 0.67 | 0.661-0.679 | 0.672 | 0.663-0.681 | 0.672 | 0.663-0.681 | 0.669 | 0.660-0.678 |
| Cancer | iAUC | 0.153 |  | 0.157 |  | 0.166 |  | 0.082 |  |
| CVD | Univariate | 0.563 | 0.554-0.572 | 0.576 | 0.566-0.585 | 0.499 | 0.490-0.508 | 0.691 | 0.683-0.699 |
| CVD | Multivariate | 0.8 | 0.793-0.807 | 0.799 | 0.792-0.806 | 0.8 | 0.792-0.807 | 0.8 | 0.792-0.807 |
| CVD | iAUC | 0.237 |  | 0.223 |  | 0.301 |  | 0.109 |  |
|  |  | **C-index** | **95%CI** | **C-index** | **95%CI** | **C-index** | **95%CI** | **C-index** | **95%CI** |
| Cancer | Univariate | 0.524 | 0.501-0.546 | 0.524 | 0.502-0.546 | 0.507 | 0.485-0.529 | 0.604 | 0.582-0.625 |
| Cancer | Multivariate | 0.699 | 0.679-0.718 | 0.7 | 0.681-0.719 | 0.7 | 0.681-0.719 | 0.698 | 0.679-0.717 |
| Cancer | iC-index | 0.175 |  | 0.176 |  | 0.193 |  | 0.094 |  |
| CVD | Univariate | 0.564 | 0.544-0.584 | 0.596 | 0.576-0.616 | 0.503 | 0.482-0.524 | 0.705 | 0.689-0.722 |
| CVD | Multivariate | 0.825 | 0.811-0.838 | 0.825 | 0.811-0.838 | 0.825 | 0.811-0.838 | 0.824 | 0.81-0.838 |
| CVD | iC-index | 0.261 |  | 0.229 |  | 0.322 |  | 0.119 |  |

Abbreviation: CVD, Cardiovascular diseases; BMI, body mass index; BSA, body surface area; BFP, body fat percentage; AUC, area under the curve; iAUC, increment AUC; iC-index, increment C-index; CI: confidence intervial.

Univariate model was the unadjusted predictive model only used one of the body measurements: BMI, Height, BSA, and BFP.

Multivariate model was the predictive model used one of the four body measurements plus age, sex, HDL-C, LDL-C, TG, FBG,and SBP.

iAUC and iC-index represents the change of predictive performance after adjusted potential confounders.


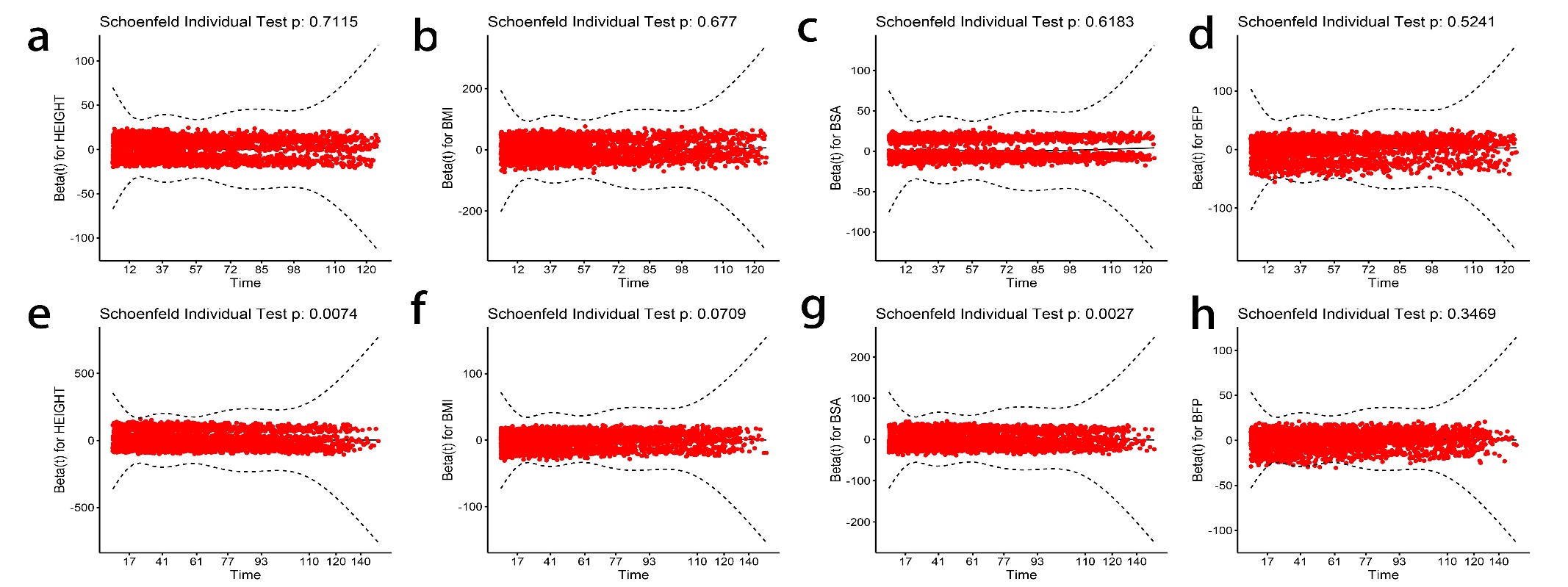


Figure S1. Schoenfeld test of proportional hazards hypothesis for BMI, Height, BSA, and BFP.

Schoenfeld's residuals should be independent of time, and if the residuals have a trend with time, evidence of violation of the proportional hazards (PH) hypothesis. On the residual graph, the horizontal axis represents time. If the residuals are evenly distributed, it indicates that the residuals are independent of time.In this graph, a-d was regression of independent variables to cancer, while e-h was regression of independent variables to CVD. Tests were performed at two levels (T1 and T3 level) for each independent variable.
